# Modelling intensive care unit capacity under different epidemiological scenarios of the COVID-19 pandemic in three Western European countries

**DOI:** 10.1093/ije/dyab034

**Published:** 2021-04-09

**Authors:** Ruth McCabe, Mara D Kont, Nora Schmit, Charles Whittaker, Alessandra Løchen, Marc Baguelin, Edward Knock, Lilith K Whittles, John Lees, Nicholas F Brazeau, Patrick G T Walker, Azra C Ghani, Neil M Ferguson, Peter J White, Christl A Donnelly, Katharina Hauck, Oliver J Watson

**Affiliations:** 1 MRC Centre for Global Infectious Disease Analysis & WHO Collaborating Centre for Infectious Disease Modelling, Abdul Latif Jameel Institute for Disease and Emergency Analytics, Imperial College London, St Mary’s Campus, Norfolk Place, London, UK; 2 Department of Statistics, University of Oxford, Oxford, UK; 3 NIHR Health Research Protection Unit in Emerging and Zoonotic Diseases, The Ronald Ross Building, University of Liverpool, Liverpool, UK; 4 NIHR Health Research Protection Unit in Modelling and Health Economics, Imperial College London, St Mary’s Campus, Norfolk Place, London, UK; 5 Modelling and Economics Unit, National Infection Service, Public Health England, London, UK

**Keywords:** COVID-19, intensive care, epidemiological modelling, hospital capacity, non-pharmaceutical interventions

## Abstract

**Background:**

The coronavirus disease 2019 (COVID-19) pandemic has placed enormous strain on intensive care units (ICUs) in Europe. Ensuring access to care, irrespective of COVID-19 status, in winter 2020–2021 is essential.

**Methods:**

An integrated model of hospital capacity planning and epidemiological projections of COVID-19 patients is used to estimate the demand for and resultant spare capacity of ICU beds, staff and ventilators under different epidemic scenarios in France, Germany and Italy across the 2020–2021 winter period. The effect of implementing lockdowns triggered by different numbers of COVID-19 patients in ICUs under varying levels of effectiveness is examined, using a ‘dual-demand’ (COVID-19 and non-COVID-19) patient model.

**Results:**

Without sufficient mitigation, we estimate that COVID-19 ICU patient numbers will exceed those seen in the first peak, resulting in substantial capacity deficits, with beds being consistently found to be the most constrained resource. Reactive lockdowns could lead to large improvements in ICU capacity during the winter season, with pressure being most effectively alleviated when lockdown is triggered early and sustained under a higher level of suppression. The success of such interventions also depends on baseline bed numbers and average non-COVID-19 patient occupancy.

**Conclusion:**

Reductions in capacity deficits under different scenarios must be weighed against the feasibility and drawbacks of further lockdowns. Careful, continuous decision-making by national policymakers will be required across the winter period 2020–2021.

**Key Messages**

- Without mitigation, the number of coronavirus disease 2019 (COVID-19) patients estimated to require intensive care in winter 2020–2021 will exceed the peak of the first wave and result in capacity deficits.
- Non-pharmaceutical interventions to suppress the transmission of SARS-CoV-2, triggered when the number of COVID-19 patients in intensive care exceeds a defined threshold, can produce substantial reductions in capacity deficits, particularly when triggered earlier and sustained under a higher level of suppression.
- Beds are consistently the most constrained intensive care unit resource.
- Mitigation of demand for intensive care must be weighed against the feasibility of suppression interventions and drawbacks of the intensity and duration of lockdown.

## Introduction

National healthcare systems are under extreme pressure due to the coronavirus disease 2019 (COVID-19) pandemic. To avoid overwhelming hospitals at the beginning of the pandemic, countries implemented stringent non-pharmaceutical interventions (NPIs) including physical distancing and national lockdowns. Although effective in reducing transmission, the economic and social costs of such interventions cast doubt on their long-term tenability.^1^ At the same time, countries increased hospital capacity to treat COVID-19 patients, with the opening of field hospitals, reorganization of health services and the cancellation of elective surgery. Nonetheless, many European countries still reported strains on intensive care unit (ICU) resources owing to a surge in demand.^2–4^

Healthcare demand is generally greatest during winter.^5^ Healthcare systems must be prepared to deal with this in addition to likely increases in numbers of COVID-19 patients requiring hospitalization in winter 2020–2021.^6–8^ The number of patients in ICUs will be heavily dependent on population behaviour and the effectiveness of NPIs. However, few studies have linked forecasts of COVID-19 healthcare demand under NPIs to national-level estimates of hospital capacity and utilization (e.g. ^9–11^).

Here, we integrate a hospital capacity framework^12^^,^^13^ with epidemiological projections of COVID-19 patients requiring ICU treatment^14^ in three European countries that have been heavily affected by the pandemic. We present a scenario-based analysis of the spare capacity of ICU beds, ventilators and staff in France, Germany and Italy over the winter period 2020–2021 under a ‘dual-demand’ (COVID-19 and non-COVID-19) patient model. We examine the effect of suppression strategies of varying effectiveness that are triggered based on different levels of ICU occupancy by COVID-19 patients.

## Methods

We estimate the spare capacity, defined as the difference between the number of patients that can be accommodated in the ICU and the number of patients requiring ICU care, of four essential resources: beds, ventilators, nurses and doctors. We apply our analysis to France, Germany and Italy.

### Parameterizing the capacity model

The capacity framework includes a ‘dual-demand’ model of care requirements incorporating demand from COVID-19 and non-COVID-19 cohorts. The former is projected under different epidemiological scenarios whereas the latter is estimated using average annual occupancy figures. The requirements of each resource are calculated per patient, with multiple data sources used to parameterize the model (Table 1).^23–33^

**Table 1:** Baseline capacity of intensive care unit (ICU) resources in France, Germany and Italy, and parameters of the capacity model with sources. Details are provided where definitions and measurement methods of ICU capacity vary between countries

Country	Variable	Value	Year of estimate	Details	Source
France	Total beds	10 640	2018	Bed ratio per 100 000 population applied to 2020 population size. Number includes ‘reanimation’ beds for adults except for severe burns and intensive care beds except for neonatology.	Organisation for Economic Cooperation and Development (OECD) Intensive Care Beds Capacity^15^ Population Division of the Department of Economic and Social Affairs of the United Nations Secretariat^16^
	Bed occupancy (%)^a^	87%	2011		Published country-wide study of ICU wards^17^
	Total doctors (FTE)	2047	2018	Data represent annual average full-time equivalents (FTEs) of doctors of various specialties working in ICU. Excludes doctors who are still in training (‘internes’).	Ministry of Health Annual Statistic of Health Establishments (SAE)^18^
	Total nurses (FTE)	12 332	2018	Data represent annual average FTEs of all nurses working in ICU (irrespective of their employer). Includes nurses with and without specialization.	Ministry of Health SAE^18^
	Total ventilators	7241	2009	Estimated by applying ratio of ventilators per ICU bed reported in 2009 to the 2018 number of ICU beds. Data represent (fixed and mobile) ventilators in ICUs only.	Survey by the Ministry of Health^19^
Germany	Total beds	28 403	2017	Bed ratio per 100 000 population applied to 2020 population size. Number includes paediatric ICU beds.	OECD Intensive Care Beds Capacity^15^ Population Division of the Department of Economic and Social Affairs of the United Nations Secretariat^16^
	Bed occupancy (%)^a^	79%	2017		Federal Statistical Office^20^
	Total doctors (FTE)	15 944	2015	Estimated by applying average ICU doctor FTE per hospital to the total number of hospitals in 2015 and scaled to 2017 assuming same increase as for ICU beds between 2015 and 2017. It is unclear whether this estimate includes junior doctors.	Report from the German Hospital Institute^21^
	Total nurses (FTE)	58 206	2015	Estimated by applying the ratio of ICU nurse FTEs per ICU beds reported in 2015 to the 2017 number of beds.	Report from the German Hospital Institute^21^
	Total ventilators	25 000	2020	Represents number of ventilators before the coronavirus disease 2019 (COVID-19) pandemic.	COVID-19 Health Systems Response Monitor, citing Ministry of Health^3^
Italy	Total beds	5200	2020	Bed ratio per 100 000 population applied to 2020 population size. Value represents the approximate number of beds in Italian ICUs at the beginning of the COVID-19 pandemic.	OECD Intensive Care Beds Capacity^15^ Population Division of the Department of Economic and Social Affairs of the United Nations Secretariat^16^
	Bed occupancy (%)^a^	48%	2017		Ministry of Health^22^
	Total doctors (FTE)	2415	2017	Data on doctors employed in ICUs were not directly available. An estimate of the headcount of ICU doctors was derived by applying the proportion of hospital doctors working in ICUs from Spain (2.9%) to the total doctors employed in hospitals in Italy. Converted to FTE using the multiplier derived from OECD physician data set.^b^	Ministry of Health^22^
	Total nurses (FTE)	5841	2017	Data on nurses employed in ICUs were not directly available. An estimate of the headcount of ICU nurses was derived by applying the proportion of hospital doctors working in ICUs from Spain (2.9%) to the total nurses employed in hospital in Italy. Converted to FTE using the multiplier derived from OECD nurse data set.^b^	Ministry of Health^22^
	Total ventilators	17 011	2017		Ministry of Health^22^
Hospital-capacity-model parameters					
France	Staff sickness	14.6%	2020	The daily population-infection risk was determined using the European Centre for Disease Prevention and Control (ECDC) 14-day cumulative number of COVID-19 cases per 100 000 and country-population estimates. This risk was inflated for healthcare workers, who are estimated to be 3.4 times more likely to be infected than the general population.	ECDC COVID-19 data^23^ Nguyen *et al.*^24^
Germany	Staff sickness	3.3%	2020		
Italy	Staff sickness	6.9%	2020		
All	Proportion of COVID-19 patients requiring ventilation	68%		The mean daily proportion of COVID-19 ICU patients using a ventilator was calculated from daily situation reports published between 1 April and 10 June 2020.	Robert Koch Institut^25^
All	Proportion of non-COVID-19 patients requiring ventilation	42%		Proportion of patients with >24 hours’ stay in ICUs on mechanical ventilation on the assessment day.	Study in German ICUs^26^
All	ICU bed-to-nurse ratio	2.5:1		Recommended or official ICU bed-to-nurse ratio in France, Germany and Italy.	Various sources^27–30^
All	ICU bed-to-doctor ratio	8:1		Recommended ICU bed-to-doctor ratio based on review of evidence from various countries.	Faculty of Intensive Care Medicine^31^^,^^32^

a Taken as the upper bound of this variable. Reductions in the deficit in capacity threshold (30% reduction in these figures to represent cancellation of electives and 0% non-COVID-19 occupancy) were considered in order to account for uncertainty surrounding the demand for care from non-COVID-19 patients this winter (see the ‘Modelled scenarios’ section).

b In the absence of country-specific data, a multiplier to convert headcounts to FTE was derived from the 2017 OECD data sets of ‘Physicians employed in hospital’ and ‘Professional nurses and midwives employed in hospitals’ by taking the median multiplier for all Western European countries (0.896 and 0.868, respectively).^33^

There is a one-to-one relationship between patients and beds. On average, only 42% of non-COVID-19 and 68% of COVID-19 patients were estimated to receive mechanical ventilation.^25^^,^^26^ Ratios of staff full-time equivalents (FTEs) per occupied bed were informed by recommended staff-to-patient ratios,^27–32^ with maxima of 2.5 ICU beds per nurse and 8 ICU beds per doctor assigned uniformly across countries. Staff availability is reduced using a staff-sickness rate to account for the impact of the virus on the workforce. These were calculated for each country using population-infection rates and a modified hazard rate for healthcare workers. This rate remains constant throughout the projection period.

Model equations and an illustration of the relationship between bed demand and deficits are provided in the Supplementary Material (Supplementary Figure 1, available as Supplementary data at *IJE* online).

### Estimating pre-pandemic baseline capacity

Baseline national ICU resources were estimated based on pre-pandemic levels. Data were derived from recent official publications from the Ministries of Health and the Organisation for Economic Cooperation and Development (OECD), where available (Table 1).^15^^,^^18^^,^^20^^,^^22^

The baseline occupancy of non-COVID-19 patients was determined using the average annual occupancy of ICU beds. Whereas admissions are often seasonal, this year, it is unclear whether winter increases will occur due to measures to reduce SARS-CoV-2 transmission and reductions in the non-COVID-19 patient care-seeking behaviour due to the pandemic.^34^^,^^35^ Therefore, figures for the average annual ICU occupancy of non-COVID-19 patients were considered the upper bound for this variable, with alternative scenarios also explored (see the ‘Modelled scenarios’ section).

### Epidemiological models

Epidemiological projections were performed by country using a previously published stochastic compartmental age-structured Susceptible-Exposed-Infected-Recovered model of SARS-CoV-2 transmission.^14^ The model estimates the number of cases going through different severity pathways of COVID-19 disease over time. The model is fitted to daily reported COVID-19 deaths from the European Centre for Disease Prevention and Control (ECDC)^36^ in a Bayesian framework (see Supplementary Material, available as Supplementary data at *IJE* online, epidemiological models for further details).^37^ For all analyses conducted, the package *squire* v0.4.34 was used.^38^

To tailor the model to capture the dynamics in mortality and ICU demand in each country, the following changes were made to the default model parameters. For France, we used the age-dependent infection fatality ratio (IFR), the probability of hospitalization and the probability of requiring an ICU bed given hospitalization estimated in a previous analysis of the first epidemic wave in France.^39^ For Italy, we used the same parameters as for France except that we incorporated a higher IFR as recently estimated from seroprevalence surveys.^40^ For Germany, no changes were made to the default model parameters, which sufficiently captured the dynamics in mortality and ICU demand. For all countries, we observed substantial triaging practices to ensure that ICU bed demand did not exceed capacity during the first peak, which we captured in the model by fitting a shorter duration of ICU stay during the first peak. The epidemiological models were assessed according to their fit to both official COVID-19 death data^41^ and ICU demand data.^25^^,^^41^

### Modelled scenarios

The calibrated model was used to project ICU demand from COVID-19 patients under different scenarios from 25 October 2020 to 1 March 2021, assuming no substantial impact from potential vaccines in this period. The spare capacity of each resource was then calculated under each of the 100 model simulations for every day of the projection period.

COVID-19 ICU demand, and by extension spare capacity, was modelled under an unmitigated (no intervention) scenario and a set of lockdown scenarios in which suppression interventions reduced SARS-CoV-2 transmission, expressed as reductions in the time-varying reproduction number R_t_. We investigated a trigger-based approach to the initiation of lockdown, implemented when the number of ICU beds required by COVID-19 patients exceeds a proportion of total baseline ICU bed provision (either 1/5, 1/4, 1/3 or 1/2). The length of each triggered lockdown was varied between 2 to 6 weeks and under two levels of suppression. First, it was assumed that subsequent lockdowns were as effective as the initial lockdown of spring 2020 in each country, defined as the lowest R_t_ estimated during this period. Second, given that the reduction in R_t_ likely to be observed in future lockdowns is ambiguous, we also explore a higher R_t_ = 0.8 during lockdowns. This may reflect the lighter suppression measures implemented, weaker adherence to such policies by the population and the emergence of more-transmissible variants of the virus that are more difficult to control. During periods of no lockdown, R_t_ is assumed to return to its estimated value on 25 October 2020. Lastly, we explored the impact of lockdowns being implemented in a non-reactive strategy, instead being introduced at the beginning of November for 2, 4 or 6 weeks before being lifted and re-implemented after 4 or 6 weeks, performed under the same two suppression R_t_ values as above.

Estimates of spare capacity over time and maximum deficits are presented as the median and 95% credible intervals (2.5^th^ and 97.5^th^ centiles) from the 100 spare capacity curves. Deficits in capacity occur when demand exceeds capacity. Under the baseline parameterization of non-COVID-19 patient ICU occupancy, the deficit threshold is defined by spare capacity falling below zero. Reductions in this threshold, resulting from decreases in non-COVID-19 occupancy, were evaluated in sensitivity analyses. Both a 30% reduction in baseline bed occupancy representing the cancellation of elective surgery^13^^,^^42^ and the removal of all non-COVID-19 patients were considered. The latter provides an upper bound on capacity rather than a realistic policy option. These alternative thresholds were calculated by subtracting the number of each resource freed under these occupancy levels from zero (the baseline threshold). Lastly, to characterize the impact of different lockdown triggers and length of lockdowns, we compare the overall mean spare capacity of beds throughout the projection period, the mean number of days with bed deficits and the mean total time spent in lockdown from the same 100 spare capacity curves.

## Results

### Baseline capacity

**Figure 1:**
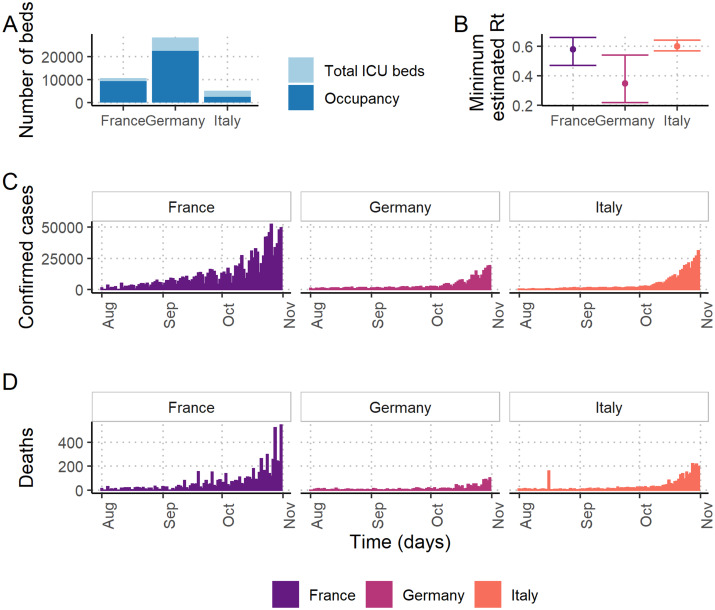
Drivers of the differences of spare capacity estimates in France, Germany and Italy. (A) The number of intensive care unit (ICU) beds and average annual non-coronavirus disease 2019 (non-COVID-19) patient occupancy at baseline. (B) The estimated minimum value of the effective reproduction number (R_t_) from the implementation of the first national lockdown (occurring in March 2020 in Italy; May 2020 in France and Germany) with 95% credible intervals. (C) The daily number of confirmed COVID-19 cases across August to November 2020 (D) The daily number of COVID-19 registered deaths across August to November 2020.

Baseline ICU capacity data were publicly available from official government publications, the OECD or academic papers, with the most recent data being from 2017–2018 (Table 1). The total number of baseline beds alongside annual average pre-pandemic non-COVID-19 occupancy is illustrated in Figure 1A.

### COVID-19 ICU demand

The calibrated epidemiological models accurately reproduced patterns of national counts of COVID-19 deaths and patients receiving ICU care in France, Germany and Italy^25^^,^^41^ (Supplementary Figure 2, available as Supplementary data at *IJE* online). In each country, the unmitigated scenarios suggest that the number of COVID-19 patients in ICUs would exceed those seen during the first epidemic wave in each country between March and June 2020 (Supplementary Figure 3, available as Supplementary data at *IJE* online). France and Italy are estimated to observe a second peak ahead of Germany, which is partly due to the greater number of confirmed cases and deaths in France and Italy recently (Figure 1C and D)^15^.

### Predictive validity

At the time of review (January 2021), it was possible to validate our simulated COVID-19 ICU demand against true numbers of COVID-19 patients in intensive care throughout 25 October–31 December 2020 (Supplementary Figure 4, available as Supplementary data at *IJE* online). During our simulation period, all three countries implemented NPIs to suppress the transmission of SARS-CoV-2.^2–4^ Consequently, in each country, observed ICU demand falls comfortably within the range of modelled scenarios, with no country following the trajectory of the unmitigated scenario.

Our simulations provide an illustration of the impact of possible mitigation scenarios as opposed to providing a prediction of epidemic progression in this period. NPIs are not implemented following an exact trigger threshold as in this study. As such, we hypothesize that differences in the timing and nature of interventions implemented, as well as observed regional variation in demand for ICU resources,^43^^,^^44^ explain the small differences between our simulations and that which was observed. For example, in France, simulated peak demand under the largest trigger threshold is of a similar magnitude to that which is observed but occurs later due to lockdown being implemented later in the simulation than in reality,^2^ whereas, in Germany, the instigation of national lockdown coincided with the date modelled for the lowest trigger threshold (mid-December),^3^ which closely followed the observed demand.

### Spare capacity in ICUs

**Figure 2:**
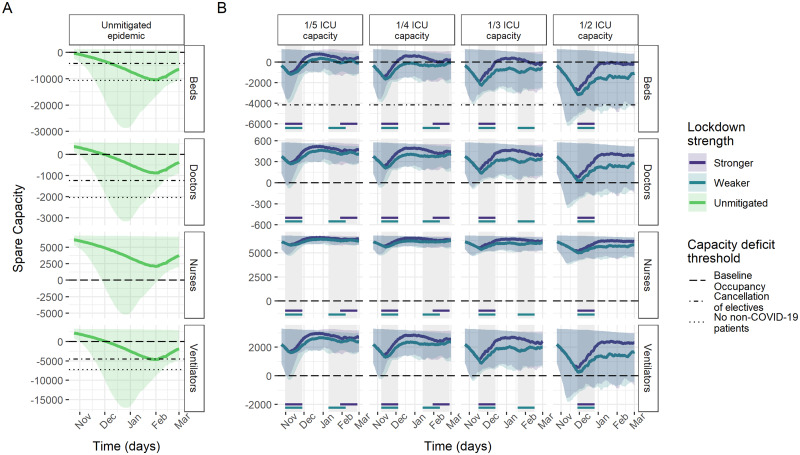
Spare capacity estimates (median; 95% credible intervals) for France. (A) The unmitigated scenario. (B) The four reactive lockdown scenarios under two different suppression levels (stronger: lockdown effective reproduction number (R_t_) = 0.58; weaker: lockdown R_t_ = 0.8) and specified lockdown length of 4 weeks. Grey-shaded areas indicate periods in which lockdowns are implemented, with horizontal coloured lines indicating the corresponding lockdown strength under which this was triggered. The dashed line (spare capacity = 0) indicates the threshold between positive spare capacity and a deficit in capacity. The dot-dashed and dotted lines indicate an effective reduction in this threshold owing to the cancellation of elective surgery and the removal of all non-coronavirus disease 2019 (non-COVID-19) patients, respectively, allowing the reallocation of resources to COVID-19 patients. ICU, intensive care unit.

**Figure 3:**
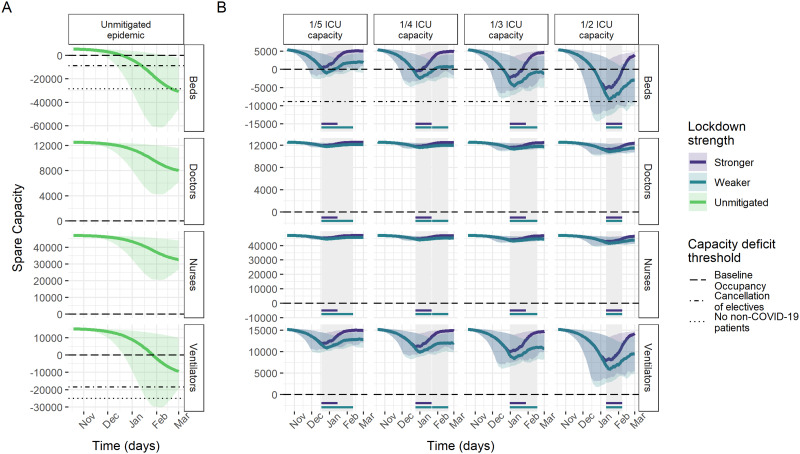
Spare capacity estimates (median; 95% credible intervals) for Germany. (A) The unmitigated scenario. (B) The four reactive lockdown scenarios under two different suppression levels (stronger: lockdown effective reproduction number (R_t_) = 0.35; weaker: lockdown R_t_ = 0.8) and specified lockdown length of 4 weeks. Grey-shaded areas indicate periods in which lockdowns are implemented, with horizontal coloured lines indicating the corresponding lockdown strength under which this was triggered. The dashed line (spare capacity = 0) indicates the threshold between positive spare capacity and a deficit in capacity. The dot-dashed and dotted lines indicate an effective reduction in this threshold owing to the cancellation of elective surgery and the removal of all non-coronavirus disease 2019 (non-COVID-19) patients, respectively, allowing the reallocation of resources to COVID-19 patients. ICU, intensive care unit.

**Figure 4:**
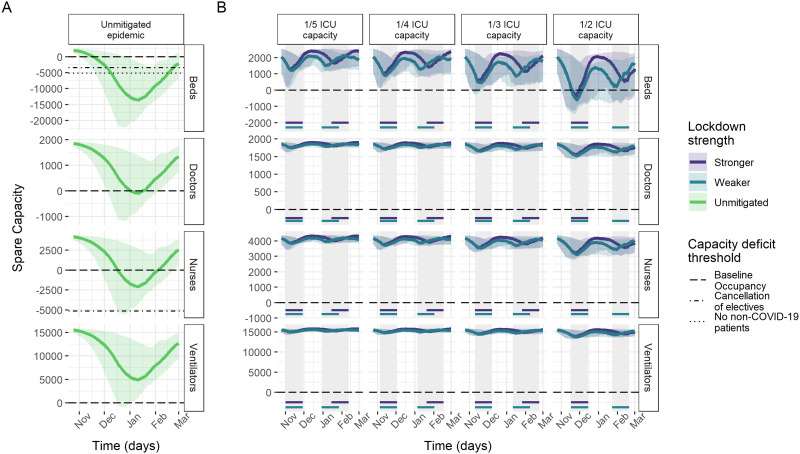
Spare capacity estimates (median; 95% credible intervals) for Italy. (A) The unmitigated scenario. (B) The four reactive lockdown scenarios under two different suppression levels (stronger: lockdown effective reproduction number (R_t_) = 0.6; weaker: lockdown effective reproduction number (R_t_) = 0.8) and specified lockdown length of 4 weeks. Grey-shaded areas indicate periods in which lockdowns are implemented, with horizontal coloured lines indicating the corresponding lockdown strength under which this was triggered. The dashed line (spare capacity = 0) indicates the threshold between positive spare capacity and a deficit in capacity. The dot-dashed and dotted lines indicate an effective reduction in this threshold owing to the cancellation of elective surgery and the removal of all non-coronavirus disease 2019 (non-COVID-19) patients, respectively, allowing the reallocation of resources to COVID-19 patients. ICU, intensive care unit.

Model results of ICU capacity constraints in France, Germany and Italy under no mitigation and different suppression scenarios sustained for 4 weeks when triggered are shown in Figures 2–4. Across countries, beds were consistently the most constrained resource. Without mitigation of the pandemic, all three countries are estimated to experience substantial shortages of ICU beds over the winter season (Table 2), with the median maximum deficits corresponding to the same number as the baseline bed capacity in Germany and France and 2.7 times the baseline bed capacity in Italy. In France and Italy, bed deficits were projected to last for almost the entire winter season, peaking in January, whereas, in Germany, they start in around December and continue to grow throughout the projection period.

**Table 2:** Median estimated maximum capacity deficit and number of days in deficit with 95% credible intervals for each country and capacity resource. The unmitigated and reactive lockdown scenarios under two suppression levels (stronger^a^: lockdown effective reproduction number (R_t_) at levels estimated during first peak; weaker: lockdown R_t_ =0.8) are presented relative to baseline occupancy. Lockdown periods are specified to last for 4 weeks.

Country	Resource	Result	Unmitigated	Stronger lockdown^a^				Weaker lockdown			
				1/5 ICU capacity	1/4 ICU capacity	1/3 ICU capacity	1/2 ICU capacity	1/5 ICU capacity	1/4 ICU capacity	1/3 ICU capacity	1/2 ICU capacity
France	Beds	Maximum capacity deficit	10 869 (0–28 902)	1217 (0–3617)	1771 (0–3641)	2808 (0–3888)	4741 (0–5963)	1278 (0–3980)	1888 (0–3906)	2915 (0–4079)	4859 (0–6327)
		Time in deficit (days)	*127 (0–127)*	*45 (0–73)*	*60 (0–83)*	*70 (0–98)*	*85 (0–125)*	*72 (0–93)*	*95 (0–118)*	*120 (0–127)*	*127 (0–127)*
	Doctors	Maximum capacity deficit	941 (0–3195)	0 (0–35)	0 (0–37)	0 (0–68)	0 (0–174)	0 (0–80)	0 (0–71)	0 (0–92)	190 (0–373)
		Time in deficit (days)	*91 (0–118)*	*0 (0–8)*	*0 (0–9)*	*0 (0–14)*	*23 (0–49)*	*0 (0–15)*	*0 (0–15)*	*0 (0–17)*	*26 (0–59)*
	Nurses	Maximum capacity deficit	0 (0–5288)	0^b^	0^b^	0^b^	0^b^	0^b^	0^b^	0^b^	0^b^
		Time in deficit (days)	*0 (0–60)*	*0^b^*	*0^b^*	*0^b^*	*0^b^*	*0^b^*	*0^b^*	*0^b^*	*0^b^*
	Ventilators	Maximum capacity deficit	4978 (0–17 240)	0 (0–47)	0 (0–63)	0 (0–231)	811 (0–1642)	0 (0–294)	0 (0–244)	0 (0–361)	892 (0–1889)
		Time in deficit (days)	*89 (0–117)*	*0 (0–4)*	*0 (0–4)*	*0 (0–9)*	*20 (0–45)*	*0 (0–13)*	*0 (0–12)*	*0 (0–16)*	*24 (0–54)*
Germany	Beds	Maximum capacity deficit	30 850 (2269–61 583)	1081 (336–2000)	2749 (1744–3923)	5491 (1991–7042)	10 805 (1991–13 132)	1598 (547–2835)	3385 (1891–4845)	6296 (1991–8476)	11 554 (1991–14 759)
		Time in deficit (days)	*72 (16–96)*	*14 (7–18)*	*24 (17–26)*	*36 (16–40)*	*51 (16–58)*	*28 (14–43)*	*41 (16–67)*	*56 (16–86)*	*71 (16–91)*
	Doctors	Maximum capacity deficit	0^b^	0^b^	0^b^	0^b^	0^b^	0^b^	0^b^	0^b^	0^b^
		Time in deficit (days)	*0^b^*	*0^b^*	*0^b^*	*0^b^*	*0^b^*	*0^b^*	*0^b^*	*0^b^*	*0^b^*
	Nurses	Maximum capacity deficit	0^b^	0^b^	0^b^	0^b^	0^b^	0^b^	0^b^	0^b^	0^b^
		Time in deficit (days)	*0^b^*	*0^b^*	*0^b^*	*0^b^*	*0^b^*	*0^b^*	*0^b^*	*0^b^*	*0^b^*
	Ventilators	Maximum capacity deficit	9458 (0–30 357)	0^b^	0^b^	0^b^	0^b^	0^b^	0^b^	0^b^	0^b^
		Time in deficit (days)	*33 (0–71)*	*0^b^*	*0^b^*	*0^b^*	*0^b^*	*0^b^*	*0^b^*	*0^b^*	*0^b^*
Italy	Beds	Maximum capacity deficit	14 402 (5777–22 822)	0^b^	0^b^	0 (0–183)	889 (379–1348)	0^b^	0^b^	0 (0–357)	995 (488–1608)
		Time in deficit (days)	*104 (64–114)*	*0^b^*	*0^b^*	*0 (0–8)*	*29 (12–37)*	*0^b^*	*0^b^*	*0 (0–14)*	*36 (15–46)*
	Doctors	Maximum capacity deficit	201 (0–1254)	0^b^	0^b^	0^b^	0^b^	0^b^	0^b^	0^b^	0^b^
		Time in deficit (days)	*23 (0–45)*	*0^b^*	*0^b^*	*0^b^*	*0^b^*	*0^b^*	*0^b^*	*0^b^*	*0^b^*
	Nurses	Maximum capacity deficit	2401 (0–5769)	0^b^	0^b^	0^b^	0^b^	0^b^	0^b^	0^b^	0^b^
		Time in deficit (days)	*50 (0–60)*	*0^b^*	*0^b^*	*0^b^*	*0^b^*	*0^b^*	*0^b^*	*0^b^*	*0^b^*
	Ventilators	Maximum capacity deficit	0 (0–1396)	0^b^	0^b^	0^b^	0^b^	0^b^	0^b^	0^b^	0^b^
		Time in deficit (days)	*0 (0–18)*	*0^b^*	*0^b^*	*0^b^*	*0^b^*	*0^b^*	*0^b^*	*0^b^*	*0^b^*

a France: R_t_ = 0.58; Germany: R_t_ = 0.35; Italy: R_t_ = 0.6.

b No deficits projected under any of the 100 simulation replicates.

ICU, intensive care unit.

Ventilators reached smaller median maximum deficits of ∼5000 and 9500 under the unmitigated scenario in France and Germany, respectively. The projections suggest no staff shortages in Germany, in contrast to a median maximum deficit of 941 doctor FTEs in France and 201 doctor FTEs and 2401 nurse FTEs in Italy (Table 2). However, reduction in baseline occupancy through the cancellation of elective surgery was estimated to be sufficient to restore the positive spare capacity of staff and ventilators (Figures 2–4). Similarly, with strong suppression measures in the lockdown scenarios, our estimates suggest that these resources generally would not reach a deficit (Table 2).

The modelled suppression scenarios highlight the large effect that reactive lockdown measures can have on mitigating shortfalls in ICU bed capacity. For a 4-week lockdown as effective as during the first peak, the magnitude and duration of shortages in ICU bed capacity varied across countries and trigger thresholds, but reductions compared with the unmitigated scenario were large throughout (Table 2 and Figures 2–4). Depending on the trigger threshold, the lockdown scenario reduced the median maximum bed deficits by between 56–89% and 65–96%, and reduced the median duration of deficits by between 33–65% and 30–81% in France and Germany, respectively. In Italy, reactive lockdowns only resulted in deficits in beds for the highest ICU trigger threshold.

Under the 1/2 ICU capacity trigger threshold lockdown scenario, remaining median maximum deficits of 4741, 10 805 and 889 beds (representing 45%, 38% and 17% of baseline bed capacity) in France, Germany and Italy, respectively, were estimated to be prevented by additional reductions in baseline ICU occupancy through the cancellation of elective surgery on average (Figures 2–4).

### Effect of varying trigger thresholds, duration and effectiveness on the impact and time in lockdown

The impact of and total time spent under a reactive lockdown were compared under different assumptions of trigger thresholds, lockdown duration and effectiveness.

**Figure 5:**
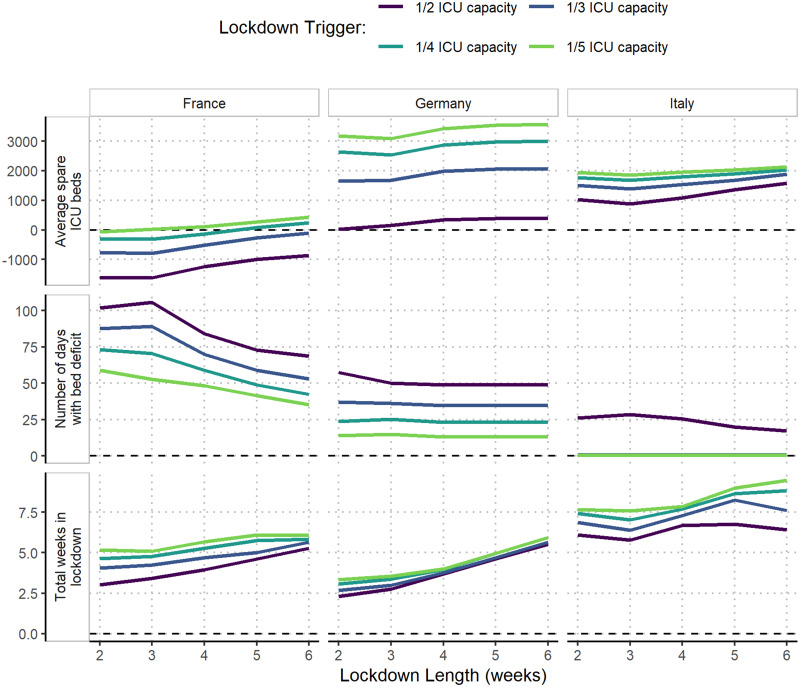
Impact of the duration and timing of lockdowns on the spare capacity of intensive care unit (ICU) beds. The effect of lockdown length on the average spare capacity of ICU beds; the number of days with a deficit in ICU beds and the total number of days spent in lockdown are shown for France, Germany and Italy under the stronger-suppression scenarios (France: effective reproduction number (R_t_) = 0.58; Germany: R_t_ = 0.35; Italy: R_t_ = 0.6). For each plot, the mean of 100 simulation repetitions over the projection period (25 October 2020–1 March 2021) is shown.

Spare ICU bed capacity varied substantially between scenarios using the highest and lowest lockdown trigger thresholds. In France and Germany, the highest trigger threshold resulted in increases of three and nine times the maximum deficits under the lowest thresholds, respectively (Table 2), and the lowest trigger threshold consistently resulted in the shortest time with a shortage of beds (Figure 5). In Italy, the lowest trigger threshold similarly resulted in the largest median spare ICU bed capacity over the projection period and bed deficits were prevented altogether under all but the 1/2 ICU capacity threshold. This difference between countries is largely explained by the substantially lower baseline occupancy of ICU beds in Italy by non-COVID patients (48%) compared with France (87%) and Germany (79%) (Figure 1A). The lower baseline occupancy affords reactive strategies more time for the impact of lockdown measures to have an effect before reaching capacity limits.

Altering the length of each lockdown by between 2 and 6 weeks resulted in minimal reductions in the maximum deficits under equivalent scenarios (Supplementary Figures 5–10 and Supplementary Tables 2–3, available as Supplementary data at *IJE* online). This is largely due to lockdowns needing to be implemented immediately in the scenarios with the lowest ICU trigger thresholds, reflecting that ICU demand was already high at the beginning of the projection period. However, the average spare ICU bed capacity tended to increase with lockdown length, notably in France, whereas the relationship between lockdown length and number of days in deficits varied by country. In Germany, the number of days of bed deficits are not dependent on lockdown length due to the particularly low level of R_t_ achieved during the first lockdown (Figures 1B and 5).

A lockdown sustained at a lower level of suppression also resulted in only small increases in the maximum bed deficits compared with the stronger lockdown for a given trigger threshold (Table 2), except for in Germany, where the lockdown during the first peak was particularly effective (Figure 1B). However, expected bed deficits persisted for longer under the weaker lockdown scenarios in all three countries, despite the total time in lockdown generally increasing under each trigger strategy (Supplementary Figure 11, available as Supplementary data at *IJE* online).

The total time spent in lockdown in France and Italy over the projection period increases slightly under lower trigger thresholds (Figure 5). In Germany, the effectiveness of the first lockdown results in a similar amount of time in lockdown under different trigger thresholds. In Italy, the similarity in the 2- and 4-week lockdown length scenarios reflects that successive lockdowns are quickly implemented in the 2-week lockdown scenario, with a 2-week lockdown unable to reduce transmission enough to reduce demand below the ICU trigger threshold. The comparatively lower assumed total ICU capacity in Italy (5200 beds) compared with France (10 640 beds) and Germany (28 403 beds) also resulted in more frequent lockdowns being implemented in Italy, with almost 6 weeks predicted to be spent in lockdown before 1 March. Lockdowns of pre-determined fixed start and end dates were generally estimated to have a similar effect on reducing deficits, but with small increases in the total amount of time spent in lockdown (Supplementary Figures 12–14, available as Supplementary data at *IJE* online).

## Discussion

In this study, we examined potential constraints of four key ICU resources in France, Germany and Italy under different epidemic scenarios over the winter of 2020–2021. The unmitigated scenarios resulted in COVID-19 ICU patient numbers exceeding those seen in the first peak, thus inducing capacity deficits. Triggered lockdown scenarios substantially reduced these deficits. Our study found that, across all epidemic scenarios, beds are consistently the most constrained ICU resource. Projections of constraints in doctors, nurses and ventilators varied across countries, but were found to be manageable through the implementation of lockdowns, as well through reductions in baseline bed occupancy, e.g. via the cancellation of elective surgery.

The results suggest that lockdowns triggered based on ICU occupancy could lead to large increases in spare bed capacity during the winter compared with no intervention, reducing deficits in all countries to lower levels that can then be managed by hospital provision interventions. Lower trigger thresholds generally minimize deficits by implementing lockdowns earlier, but their impact is dependent on baseline ICU bed numbers and average non-COVID-19 patient occupancy. For example, Italy, with a lower average occupancy, can accommodate greater demand from COVID-19 patients relative to the total ICU bed capacity. For a given trigger threshold, increasing the length of lockdown only provides small decreases in the number of days in deficits. On the other hand, a lockdown less effective than the first peak reduces deficits compared with the unmitigated scenario but could also lead to an increase in the amount of time spent in lockdown and the requirement of a lower trigger threshold compared with a stronger lockdown. Our results highlight the dependencies between these metrics, suggesting that absolute benefits of different strategies must be weighed against the feasibility and drawbacks of an increased amount of time spent in lockdown.

Our study integrates two critical frameworks of significance in the control of the pandemic: hospital capacity estimation and epidemiological simulations. Whereas previous studies have used epidemiological modelling to project ICU demand, data on hospital capacity failed to consider key ICU resources other than beds and the dependencies between them.^12^ Further strengths of this study include the use of a dual-demand model considering changes in demand for ICU care of both COVID-19 and non-COVID-19 patients, and the incorporation of COVID-19-related staff sickness that has shown to result in substantial additional constraints.^45^ Though countries have differing definitions of intensive care, our results also provide insights into how requirements for ICU capacity management may vary between countries with different healthcare systems and epidemic trajectories. Specifically, this allows policymakers to consider how to combine interventions to alleviate strain on hospital capacity effectively via the use of NPIs, by reducing the number of non-COVID-19 patients and increasing ICU capacity through the use of supply-side provision interventions (see Supplementary Material, available as Supplementary data at *IJE* online, for further discussion).

There are some limitations of this study. First, data were sometimes missing or of low quality. Due to poor documentation at the national level, it was not possible to quantify the expansion of hospital capacity during the first peak of COVID-19 patients. Capacity deficits may be overestimated, although many of the implemented hospital provision interventions were temporary. Data from the first peaks in spring 2020 used to parameterize the model, e.g. ventilation requirements, may have changed due to changes in clinical practice. For example, the use of dexamethasone for treating individuals receiving oxygen has been shown to decrease COVID-19 mortality.^46^ Data on the use of dexamethasone over time in each country are lacking; the resultant reduction of IFR would lead to an increased ICU demand in order to reproduce the observed mortality. This could be the explanation for our slight underestimation of ICU demand at the beginning of the second wave (Supplementary Figures 2 and 4, available as Supplementary data at *IJE* online). Second, modelled estimates of the spare capacity of nurses and doctors are likely to be uncertain as ward-based bed-to-staff ratios have previously been shown to be inconsistent in approximating national staffing requirements^47^^,^^48^ and there is no single recommended methodological standard for staff ratios across the countries.^49^ However, our unmitigated scenario results broadly align with a recent study analysing healthcare pressure in Europe due to the COVID-19 pandemic.^50^ Third, the model does not account for cohorting of COVID-19 and non-COVID-19 patients within hospitals, which likely reduces available resources, but this will vary between hospitals and is beyond the scope of this analysis. Fourth, whereas R_t_ may not remain constant during lockdown periods, we made this simplifying assumption to avoid having a confusing number of alternative scenarios. This means that we did not account for the emergence of more-transmissible variants of SARS-CoV-2 during the simulation period, which may increase pressure on health services despite implemented lockdown measures. However, our scenarios broadly encompass true COVID-19 ICU demand within each country in November and December 2020 (Supplementary Figure 4, available as Supplementary data at *IJE* online). Fifth, the comparison between different lockdown triggers and lengths is limited by having a fixed end date for comparison (1 March 2021). Consequently, the timing of lockdowns within this evaluation period leads to non-monotonic relationship between lockdown length and the spare capacity of ICU beds. Lastly, we do not consider the impact of vaccines. COVID-19 vaccines were approved for use across Europe in December 2020.^51^ However, over the time period that we consider, vaccination will have a very small effect, due to the time taken for a significant proportion to receive the vaccine and then develop immune protection.

While the trajectory of the COVID-19 pandemic over winter is unknown, our findings suggest that a combination of strategies will be required to overcome potential ICU capacity deficits and ensure the treatment of all patients, regardless of COVID-19 status, in France, Germany and Italy. Although this analysis focused on these three countries, similar questions surrounding the required winter interventions must now be answered across Europe, with substantial second waves being observed across the continent, which have eclipsed the first wave in several countries. The large trade-offs inherent in each strategy should not be underestimated and continuous decision-making by national policymakers will be required across the winter period 2020–2021.

## Supplementary Data

Supplementary Data are available at *IJE* online.

## Author Contributions

R.M., M.D.K. and N.S. are joint first authors and contributed equally to the conception and design of the study along with O.J.W., K.H. and P.J.W. O.J.W., C.W., P.G.T.W. and A.G. developed the COVID-19 simulation model with input from N.M.F., M.B., E.K., L.W. and J.L. N.F.B. and O.J.W. performed COVID-19 model calibration. O.J.W. and R.M. conducted the simulations and analysis. N.S. and A.L. collated baseline ICU data that were verified by M.D.K. and R.M. R.M. wrote the first draft with input from M.D.K., N.S., K.H. and O.J.W. All authors contributed to redrafting.

## Funding

This work was supported by the MRC Centre for Global Infectious Disease Analysis (grant number MR/R015600/1 to A.C.G., N.M.F., P.J.W., C.D. and K.H.), which is jointly funded by the UK Medical Research Council (MRC) and the UK Foreign, Commonwealth & Development Office (FCDO), under the MRC/FCDO Concordat agreement and is also part of the EDCTP2 programme supported by the European Union (EU); by Community Jameel (to R.M., K.H. and N.M.F.); by the Imperial College Medical Research Council Doctoral Training Partnership (to M.D.K., N.S. and C.W.); by the National Institute for Health Research (NIHR) HPRU in Modelling and Health Economics, a partnership between Public Health England (PHE), Imperial College London and LSHTM (grant number NIHR200908 to N.M.F., P.J.W. and K.H.); by the NIHR HPRU in Emerging and Zoonotic Infections, a partnership between PHE, University of Oxford, University of Liverpool and Liverpool School of Tropical Medicine (grant number NIHR200907 to R.M. and C.A.D.); and by the Wellcome Trust and FCDO (to O.J.W., P.G.T.W., A.C.G. and N.M.F).

## Supplementary Material

dyab034_Supplementary_Data
