# Supplementary material for: Modelling intensive care unit capacity under different epidemiological scenarios of the COVID-19 pandemic in three Western European countries
Source: Int J Epidemiol. 2021 Apr 9;50(3):753–67. doi: 10.1093/ije/dyab034 (PMC8083295; doi:10.1093/ije/dyab034)
Supplement: dyab034_Supplementary_Data [file dyab034_supplementary_data.docx]

## Supplementary Material

### Calculation of spare capacity

The calculations underpinning spare capacity estimates as set out in *Methods* are described mathematically below. An overview of the notation used is provided in Supplementary Table S1, while Supplementary Figure S1 provides an illustration of the relationship between intensive care unit (ICU) bed demand and capacity.

**Supplementary Table S1: Notation used in spare capacity equations.**

| **Subscripts** | |
| --- | --- |
| $c$ | Refers to capacity variable (beds, nurses, doctors, ventilators) |
| $t$ | Refers to the day in the projection period |
| $r$ | Refers to the simulation replicate (ranging from 1 to 100) |
| **Variables** | |
| $B$ | Total number of beds |
| $P_{t.r}^{COVID}$ | Expected bed occupancy of coronavirus disease 2019 (COVID-19) patients at time $t$ under replicate $r$ |
| $P^{non-COVID}$ | Average bed occupancy for non-COVID-19 patients |
| $V$ | The number of ventilators |
| $N$ | Total nurse FTE |
| $D$ | Total doctor FTE |
| Model Parameters | |
| ${pV}^{COVID}$ | Percentage of COVID-19 patients requiring a ventilator |
| ${pV}^{non-COVID}$ | Percentage of non-COVID-19 patients requiring a ventilator |
| $b_{N}$ | Maximum number of ICU beds that a nurse could safely look after |
| $b_{D}$ | Maximum number of ICU beds that a doctor could safely look after |
| $s_{N}$ | Rate of COVID-19 related nurse sickness or absence |
| $s_{D}$ | Rate of COVID-19 related doctor sickness or absence |

The primary outcome was the spare capacity resource (beds, doctors, nurses and ventilators) at each point of the projection period, which was calculated as:

$${Spare capacity}_{c,t,r}={Resource available}_{c,t,r}-{Resource needed}_{c,t,r}$$

$$for c\in\left\{ beds, doctors,nurses, ventilators \right\}$$

$$for t\in\{projection period\}$$

$$for r\in\{1,\ldots,100\}$$

For the four resources, this equation translates to the following:

$${Spare capacity of beds}_{t,r}=B-{Beds needed}_{t,r}$$

where: ${Beds needed}_{t,r}=P_{t,r}^{COVID}+P^{non-COVID}$

$${Spare capacity of doctors}_{t}=(\left( 1-s_{D} \right)\times D)-\frac{{Beds needed}_{t,r}}{b_{D}}$$

$${Spare capacity of nurses}_{t,r}=(\left( 1-s_{N} \right)\times N)-\frac{{Beds needed}_{t,r}}{b_{N}}$$

$${Spare capacity of ventilators}_{t,r}=V-({pV}^{COVID}\times P_{t,r}^{COVID}+ {pV}^{non-COVID}\times P^{non-COVID} )$$

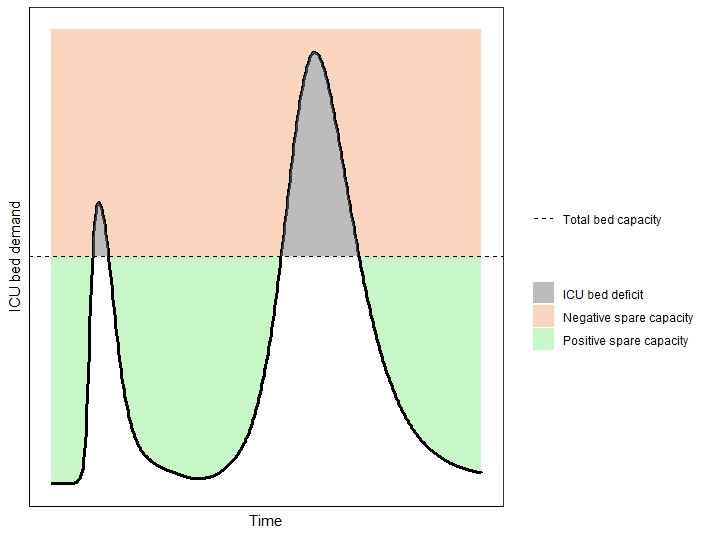


**Supplementary Figure S1: Graphical illustration of the relationship between bed demand and capacity.** The solid black line indicates demand for intensive care from coronavirus disease 2019 (COVID-19) and non-COVID-19 patients while the dashed black line indicates the total supply of beds available. Demand falling below this line (green) results in positive spare capacity of beds to treat further patients, whereas demand falling above this line (red) indicates a negative spare capacity (capacity deficit) with demand outstripping supply. The magnitudes and duration of intensive care unit (ICU) capacity deficits are captured by the grey shaded area.

### Epidemiological models

We provide an overview of the model fitting process, before detailing model changes made to tailor the model to the three European countries explored. In overview, when fitting the model, we consider the time series of deaths from coronavirus disease 2019 (COVID-19), D_t_, as a partially observed Markov process, which is given by:

D_t_ = NB(µ, [σ](https://en.wiktionary.org/wiki/%CF%83))

where NB is the Negative Binomial distribution, with mean µ and standard deviation σ. σ can be expressed as √(µ + µ^2^/*r*), where *r* is the dispersion parameter and assumed to be equal to two to account for overdispersion. The model is fit to D_t_ by allowing four parameters to vary: the start date of the epidemic, t_0_; the initial reproduction number in the absence of interventions, R_0_; the effect size of mobility sourced from the Google Mobility Reports^1^ on transmission, Mα; and the effect size of mobility on transmission after mobility increases from its minimum, Mω, which acts on increases relative to this minimum. In addition, we include pseudo-random walk parameters to reflect changes in human behaviour over time, introduced one week after the minimum in mobility, which capture changes in transmission that are independent to mobility. We fit the model to the data using a Metropolis-Hastings Markov Chain Monte Carlo (MCMC)-based sampling scheme. Model projections were subsequently created by drawing 100 parameter sets from the posterior parameter space from model fitting.

Supplementary Figure S2 shows the epidemic fit of COVID-19 deaths and ICU fits compared to the observed data for France, Germany and Italy.

Supplementary Figure S3 shows the unmitigated scenarios for France, Germany, and Italy.


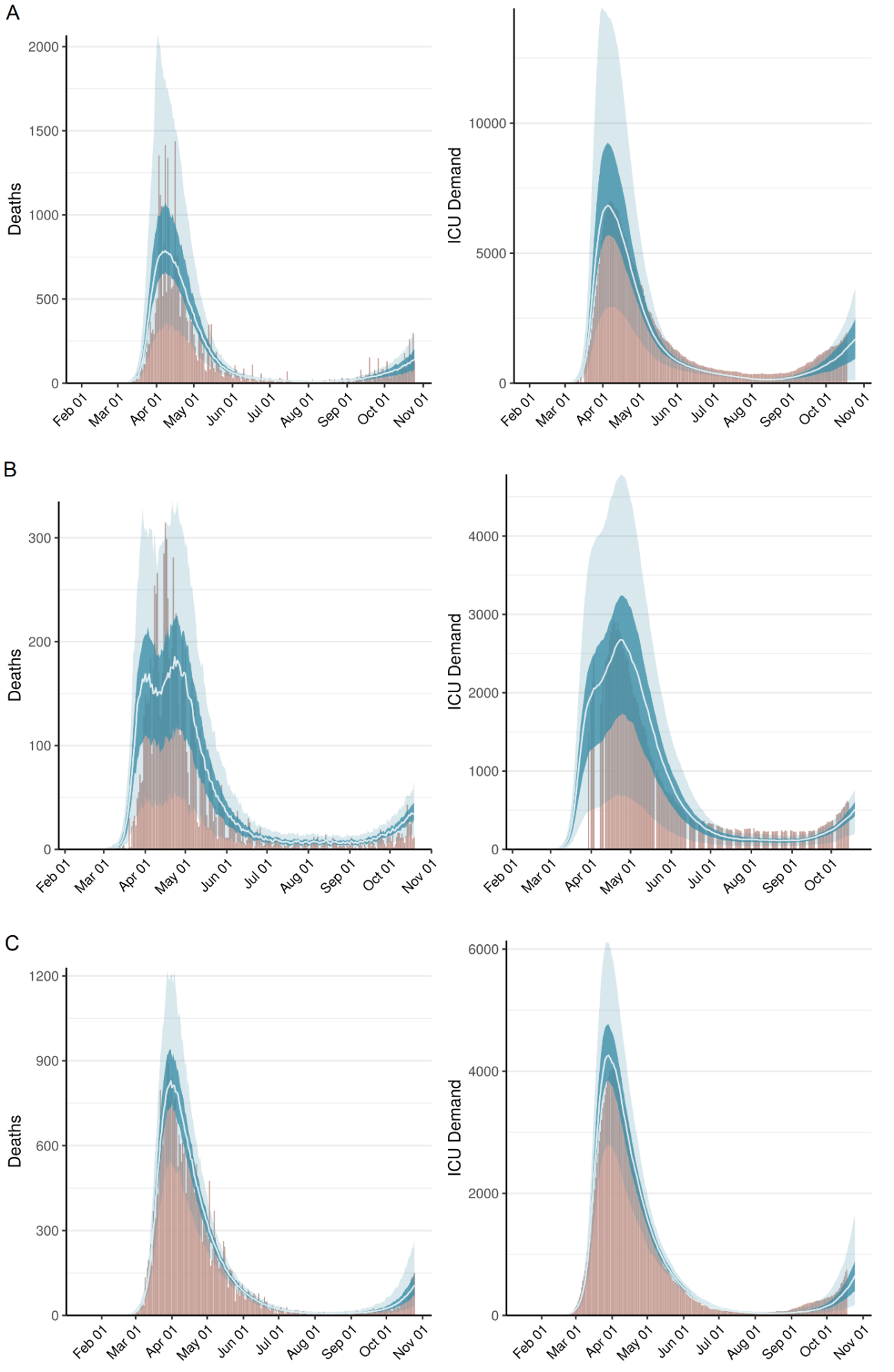


**Supplementary Figure S2: Calibrated epidemiological model to daily deaths and intensive care unit (ICU) demand.** Model estimated deaths (left) and daily number of COVID-19 patients in ICU (right) is shown in blue (dark blue 50% interquartile range, light blue 95% quantile), with reported deaths and ICU demand shown in red for (A) France^2^, (B) Germany^3^, and (C) Italy^2^.

**
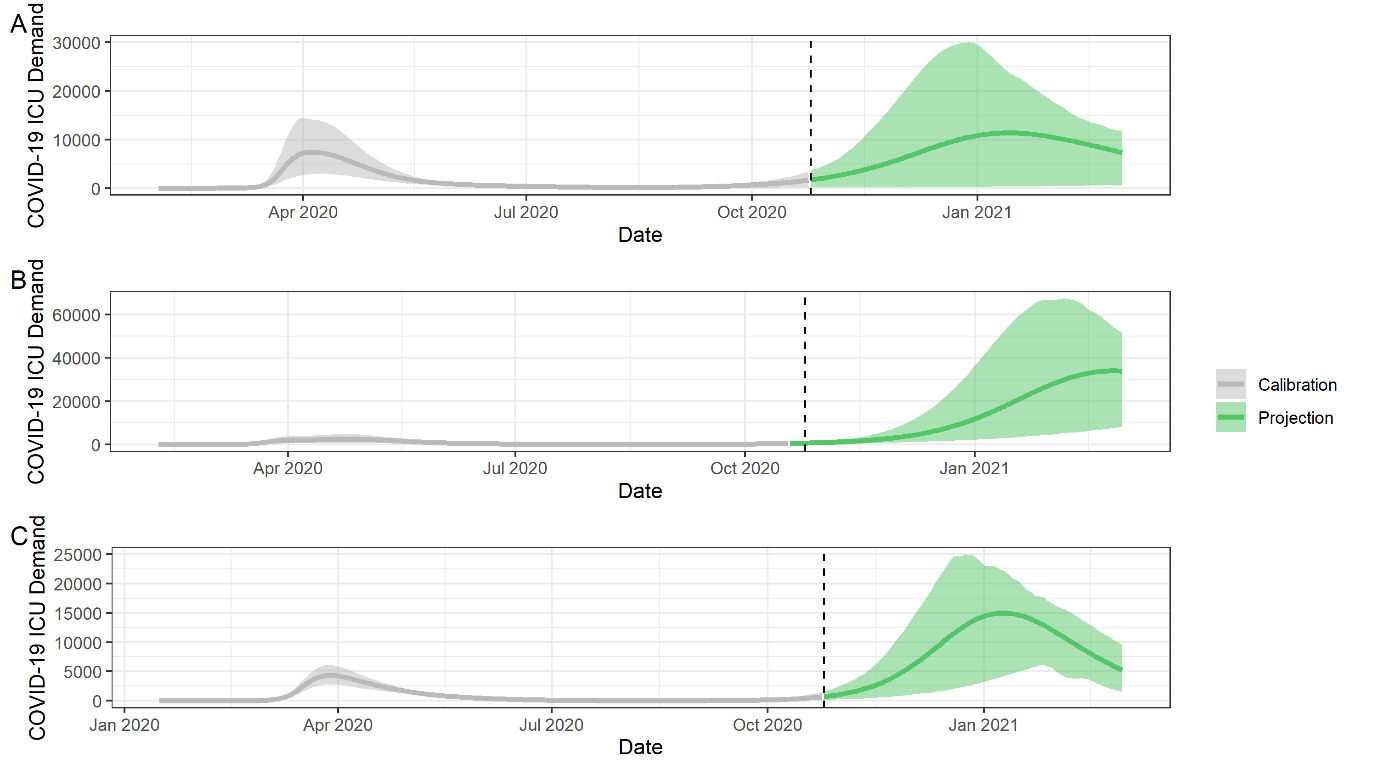
****Supplementary Figure S3: Unmitigated epidemic scenarios (median; 95% credible intervals).** (A) France, (B) Germany, and (C) Italy under current estimated effective reproduction number (R_t_) values. The projection period indicated in green with grey showing the epidemic fit.

#### Predictive validity


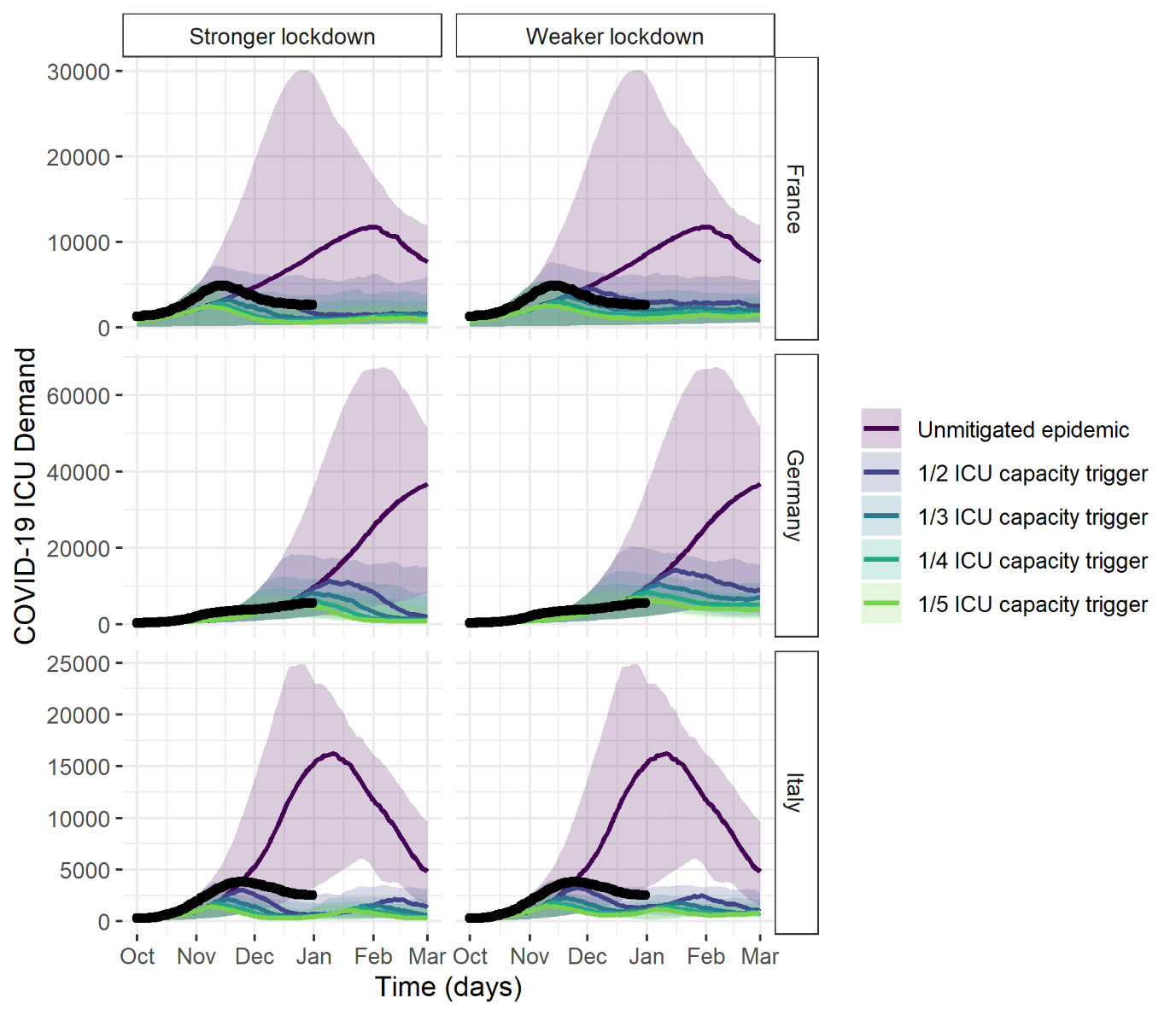
Supplementary Figure S4 shows simulated COVID-19 ICU demand throughout October 2020 – March 2021 under different scenarios compared to observed demand (up to 31^st^ December 2020).

**Supplementary Figure S4: Observed vs. simulated coronavirus disease 2019 (COVID-19) intensive care unit (ICU) demand.** Simulated demand (median; 95% credible intervals) for intensive care from COVID-19 patients in each country under different epidemiological scenarios and lockdown strengths are shown by coloured lines and shaded areas. Black dots show observed ICU demand from 1^st^ October 2020 – 31^st^ December 2020 for France^2^, Germany^3^ and Italy^2^.

### Spare capacity estimates per country and lockdown scenario

#### Two-week lockdown scenarios

Supplementary Figures S5-S7 show median spare capacity estimates and 95% credible intervals under the two levels of suppression for different lockdown triggers, assuming a lockdown length of two weeks for France, Germany, and Italy, respectively. Supplementary Table S2 shows the maximum observed deficits under each trigger threshold for each suppression level and country. These are analogous to Figures 2-4 and Table 2.


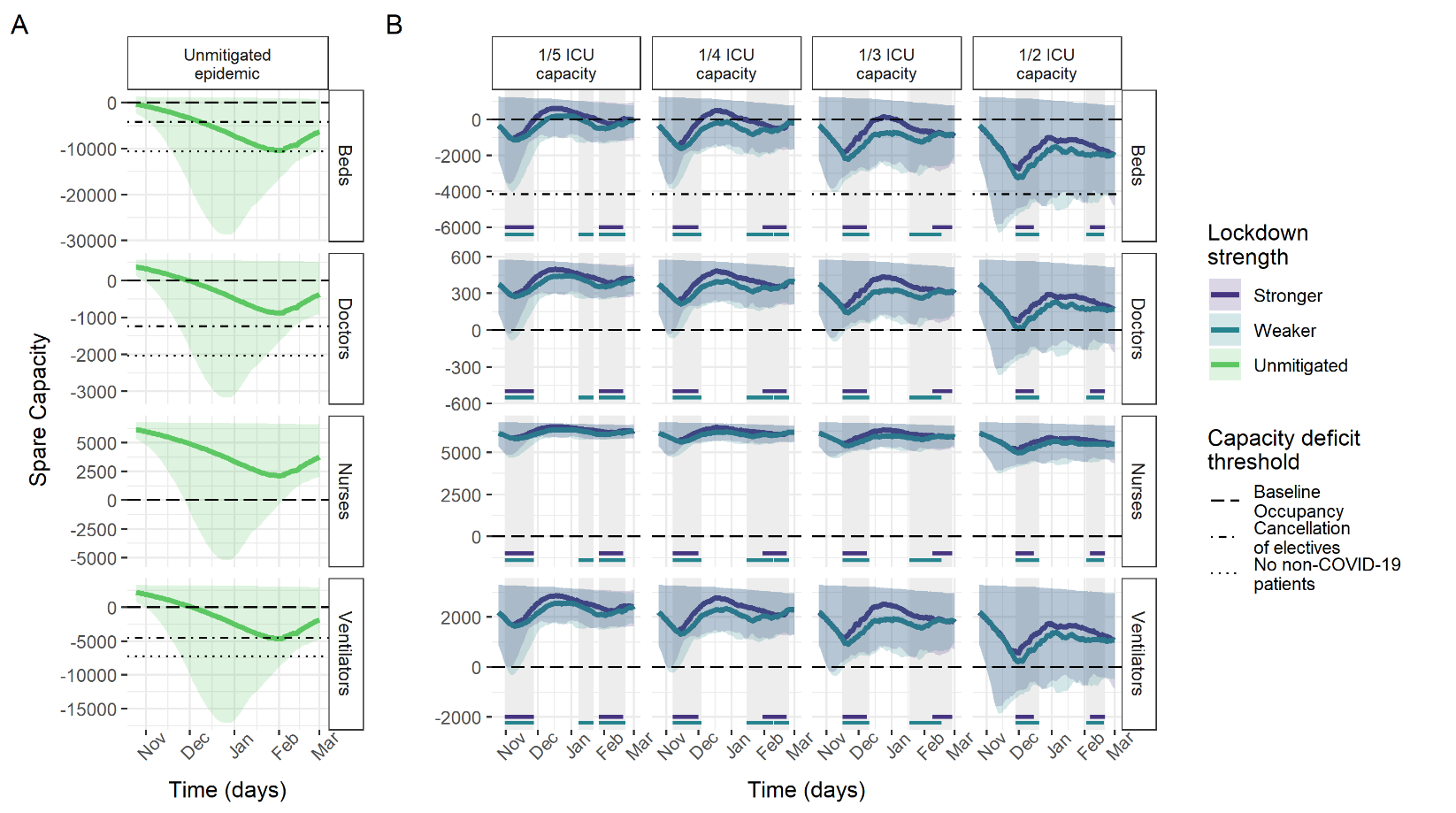


**Supplementary Figure S5: Spare capacity estimates (median; 95% credible intervals) for France.** (A) The unmitigated scenario. (B) The four reactive lockdown scenarios under two different suppression levels (stronger: lockdown effective reproduction number (R_t_) = 0.58; weaker: lockdown R_t_ = 0.8) and specified lockdown length of two weeks. Grey shaded areas indicate periods in which lockdowns are implemented, with horizontal coloured lines indicating the corresponding lockdown strength under which this was triggered. The dashed line (spare capacity = 0) indicates the threshold between positive spare capacity and a deficit in capacity. The dot-dashed and dotted lines indicate an effective reduction in this threshold owing to the cancellation of elective surgery and the removal of all non-coronavirus disease 2019 (non-COVID-19) patients respectively, allowing the reallocation of resources to COVID-19 patients.


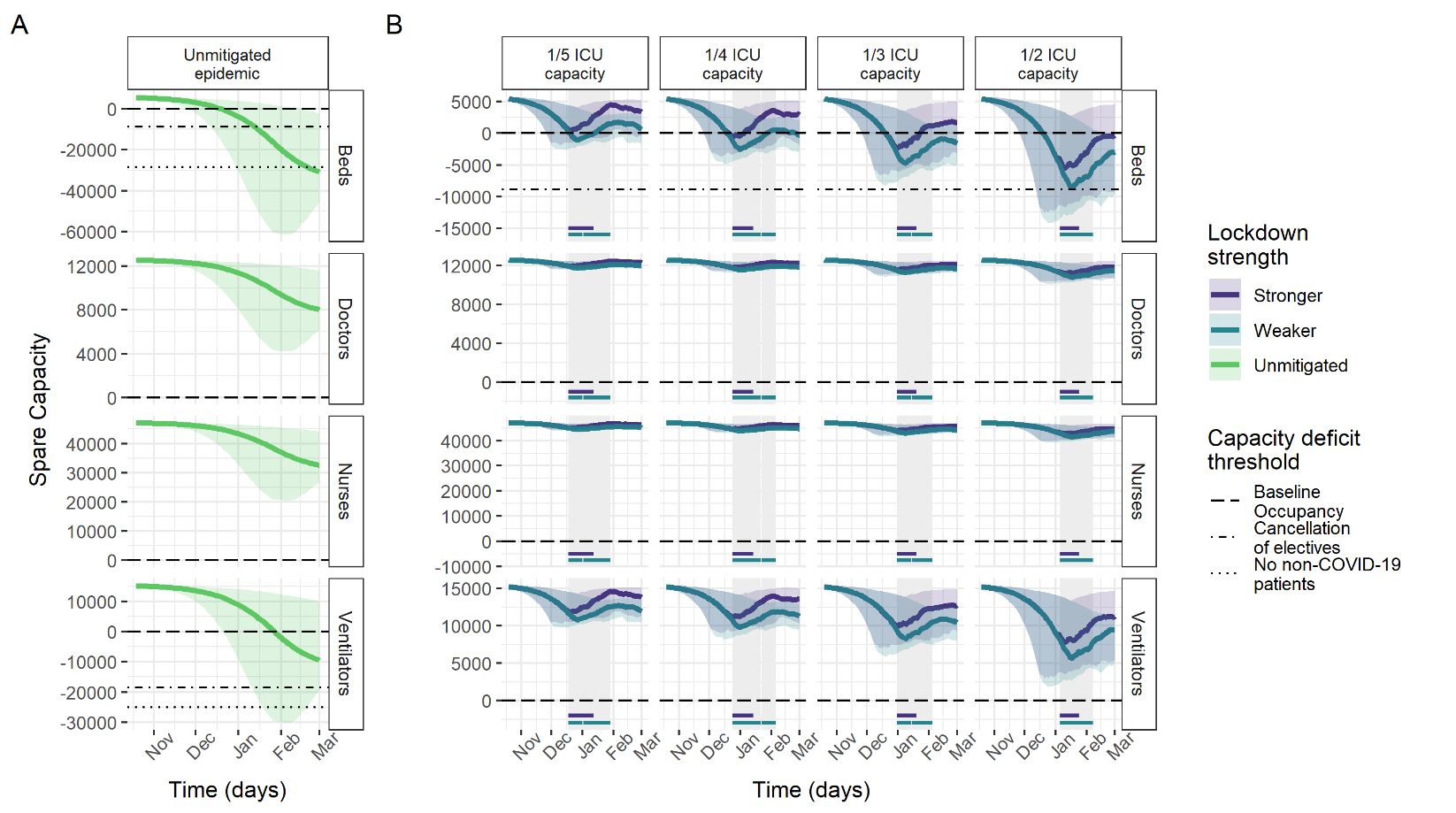


**Supplementary Figure S6: Spare capacity estimates (median; 95% credible intervals) for Germany.** (A) The unmitigated scenario. (B) The four reactive lockdown scenarios under two different suppression levels (stronger: lockdown effective reproduction number (R_t_) = 0.35; weaker: lockdown R_t_ = 0.8) and specified lockdown length of two weeks. Grey shaded areas indicate periods in which lockdowns are implemented, with horizontal coloured lines indicating the corresponding lockdown strength under which this was triggered. The dashed line (spare capacity = 0) indicates the threshold between positive spare capacity and a deficit in capacity. The dot-dashed and dotted lines indicate an effective reduction in this threshold owing to the cancellation of elective surgery and the removal of all non-coronavirus disease 2019 (non-COVID-19) patients respectively, allowing the reallocation of resources to COVID-19 patients.


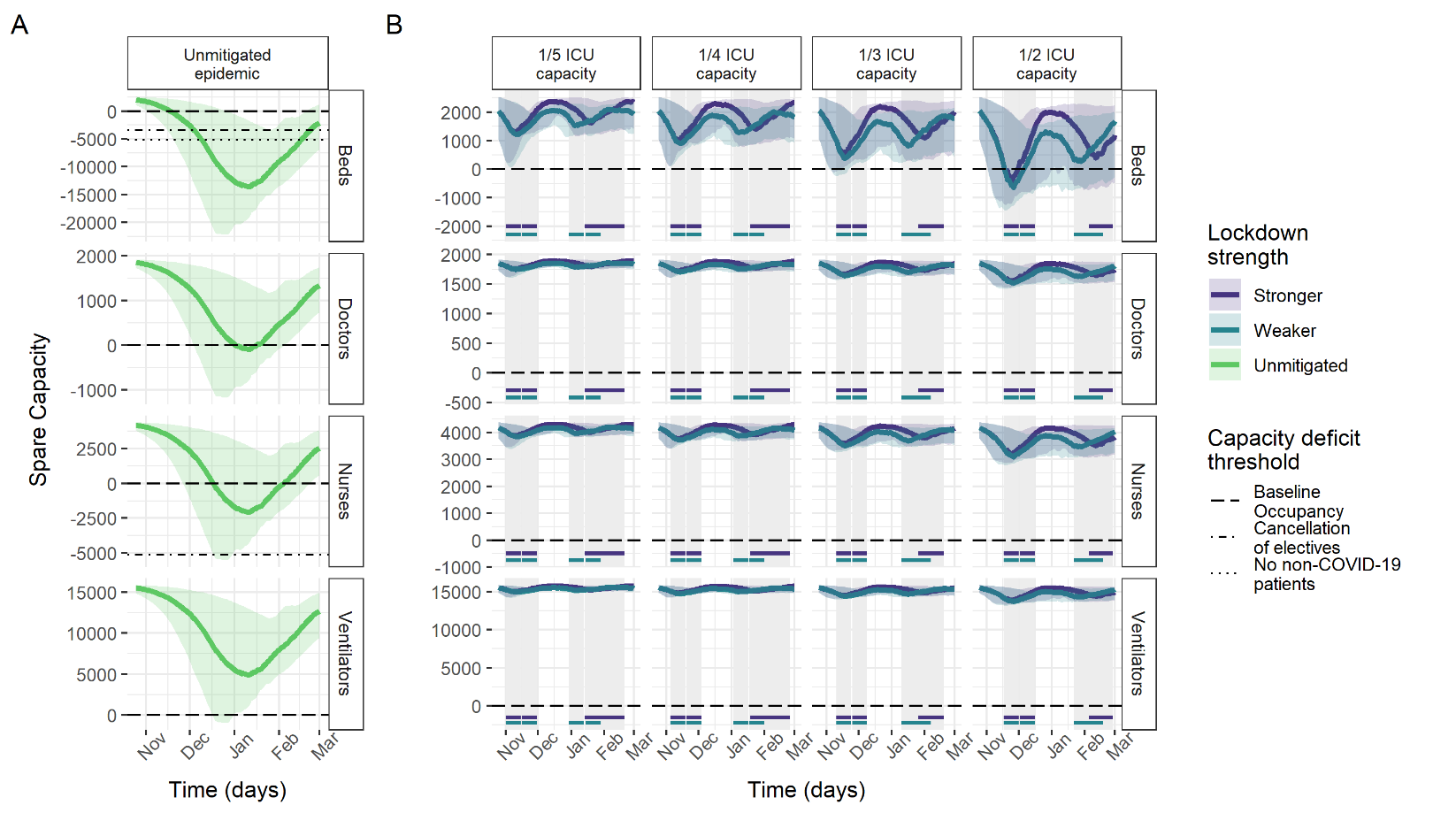


**Supplementary Figure S7: Spare capacity estimates (median; 95% credible intervals) for Italy.** (A) The unmitigated scenario. (B) The four reactive lockdown scenarios under two different suppression levels (stronger: lockdown effective reproduction number (R_t_) = 0.6; weaker: lockdown R_t_ = 0.8) and specified lockdown length of two weeks. Grey shaded areas indicate periods in which lockdowns are implemented, with horizontal coloured lines indicating the corresponding lockdown strength under which this was triggered. The dashed line (spare capacity = 0) indicates the threshold between positive spare capacity and a deficit in capacity. The dot-dashed and dotted lines indicate an effective reduction in this threshold owing to the cancellation of elective surgery and the removal of all non-coronavirus disease 2019 (non-COVID-19) patients respectively, allowing the reallocation of resources to COVID-19 patients.

**Supplementary Table S2: Median estimated maximum capacity deficit and number of days in deficit with 95% credible intervals for each country and capacity resource.** The unmitigated and reactive lockdown scenarios under two suppression levels (stronger^a^: lockdown effective reproduction number (R_t_) at levels estimated during first peak; weaker: lockdown R_t_ =0.8) are presented relative to baseline occupancy. Lockdown periods are specified to last for two weeks.

| Country | Resource | Result | Stronger lockdown^a^ | | | | Weaker lockdown | | | |
| --- | --- | --- | --- | --- | --- | --- | --- | --- | --- | --- |
|  |  |  | 1/5 ICU capacity | 1/4 ICU capacity | 1/3 ICU capacity | 1/2 ICU capacity | 1/5 ICU capacity | 1/4 ICU capacity | 1/3 ICU capacity | 1/2 ICU capacity |
| France | Beds | Maximum capacity deficit | 1209  (0-3591) | 1800  (0-3628) | 2767  (0-3898) | 4753  (0-6022) | 1287  (0-4018) | 1866  (0-3884) | 2909  (0-4078) | 4799  (0-6411) |
|  |  | Time in deficit (days) | *64*  *(0-85)* | *76*  *(0-121)* | *0*  *(0-13)* | *109*  *(0-127)* | *87*  *(0-108)* | *103*  *(0-126)* | *126*  *(0-127)* | *127*  *(0-127)* |
|  | Doctors | Maximum capacity deficit | 0  (0-31) | 0  (0-35) | 0  (0-70) | 176  (0-335) | 0  (0-85) | 0  (0-68) | 0  (0-92) | 182  (0-384) |
|  |  | Time in deficit (days) | *0*  *(0-8)* | *0*  *(0-9)* | *0*  *(0-13)* | *23*  *(0-47)* | *0*  *(0-16)* | *0*  *(0-15)* | *0*  *(0-19)* | *27*  *(0-62)* |
|  | Nurses | Maximum capacity deficit | 0^b^ | 0^b^ | 0^b^ | 0^b^ | 0^b^ | 0^b^ | 0^b^ | 0^b^ |
|  |  | Time in deficit (days) | *0^b^* | *0^b^* | *0^b^* | *0^b^* | *0^b^* | *0^b^* | *0^b^* | *0^b^* |
|  | Ventilators | Maximum capacity deficit | 0  (0-29) | 0  (0-55) | 0  (0-238) | 819  (0-1682) | 0  (0-320) | 0  (0-229) | 0  (0-360) | 850  (0-1947) |
|  |  | Time in deficit (days) | *0*  *(0-3)* | *0*  *(0-5)* | *0*  *(0-9)* | *20*  *(0-43)* | *0*  *(0-13)* | *0*  *(0-12)* | *0*  *(0-16)* | *24*  *(0-58)* |
| Germany | Beds | Maximum capacity deficit | 1098  (280-1948) | 2731  (1805-3922) | 5484  (1991-7052) | 10,787  (1991-13,280) | 1657  (509-2819) | 3383  (1926-4946) | 6276  (1991-8526) | 11,674  (1991-14,625) |
|  |  | Time in deficit (days) | *14*  *(7-23)* | *24*  *(18-29)* | *37*  *(16-51)* | *59*  *(16-82)* | *29*  *(12-56)* | *42*  *(15-71)* | *62*  *(16-86)* | *72*  *(16-92)* |
|  | Doctors | Maximum capacity deficit | 0^b^ | 0^b^ | 0^b^ | 0^b^ | 0^b^ | 0^b^ | 0^b^ | 0^b^ |
|  |  | Time in deficit (days) | 0^b^ | 0^b^ | 0^b^ | 0^b^ | 0^b^ | 0^b^ | 0^b^ | 0^b^ |
|  | Nurses | Maximum capacity deficit | *0^b^* | *0^b^* | *0^b^* | *0^b^* | *0^b^* | *0^b^* | *0^b^* | *0^b^* |
|  |  | Time in deficit (days) | 0^b^ | 0^b^ | 0^b^ | 0^b^ | 0^b^ | 0^b^ | 0^b^ | 0^b^ |
|  | Ventilators | Maximum capacity deficit | 0^b^ | 0^b^ | 0^b^ | 0^b^ | 0^b^ | 0^b^ | 0^b^ | 0^b^ |
| Germany | Ventilators | Time in deficit (days) | *0^b^* | *0^b^* | *0^b^* | *0^b^* | *0^b^* | *0^b^* | *0^b^* | *0^b^* |
| Italy | Beds | Maximum capacity deficit | 0^b^ | 0^b^ | *0*  *(0-222)* | 892  (370-1413) | 0^b^ | 0^b^ | 0  (0-301) | 1023  (506-1585) |
|  |  | Time in deficit (days) | *0^b^* | *0^b^* | *0*  *(0-8)* | *29*  *(11-38)* | *0^b^* | *0^b^* | *0*  *(0-13)* | *36*  *(15-46)* |
|  | Doctors | Maximum capacity deficit | 0^b^ | 0^b^ | 0^b^ | 0^b^ | 0^b^ | 0^b^ | 0^b^ | 0^b^ |
|  |  | Time in deficit (days) | *0^b^* | *0^b^* | *0^b^* | *0^b^* | *0^b^* | *0^b^* | *0^b^* | *0^b^* |
|  | Nurses | Maximum capacity deficit | 0^b^ | 0^b^ | 0^b^ | 0^b^ | 0^b^ | 0^b^ | 0^b^ | 0^b^ |
|  |  | Time in deficit (days) | *0^b^* | *0^b^* | *0^b^* | *0^b^* | *0^b^* | *0^b^* | *0^b^* | *0^b^* |
|  | Ventilators | Maximum capacity deficit | 0^b^ | 0^b^ | 0^b^ | 0^b^ | 0^b^ | 0^b^ | 0^b^ | 0^b^ |
|  |  | Time in deficit (days) | *0^b^* | *0^b^* | *0^b^* | *0^b^* | *0^b^* | *0^b^* | *0^b^* | *0^b^* |

^a^ France: R_t_ = 0.58; Germany: R_t_ = 0.35; Italy: R_t_ = 0.6.

^b^ No deficits projected under any of the 100 simulation replicates.

#### Six-week lockdown scenarios

Supplementary Figures S8-S10 show median spare capacity estimates and 95% credible intervals under the two levels of suppression for different lockdown triggers, assuming a lockdown length of six weeks for France, Germany, and Italy, respectively. Supplementary Table S3 shows the maximum observed deficits under each trigger threshold for each suppression level and country. These are analogous to Figures 2-4 and Table 2.


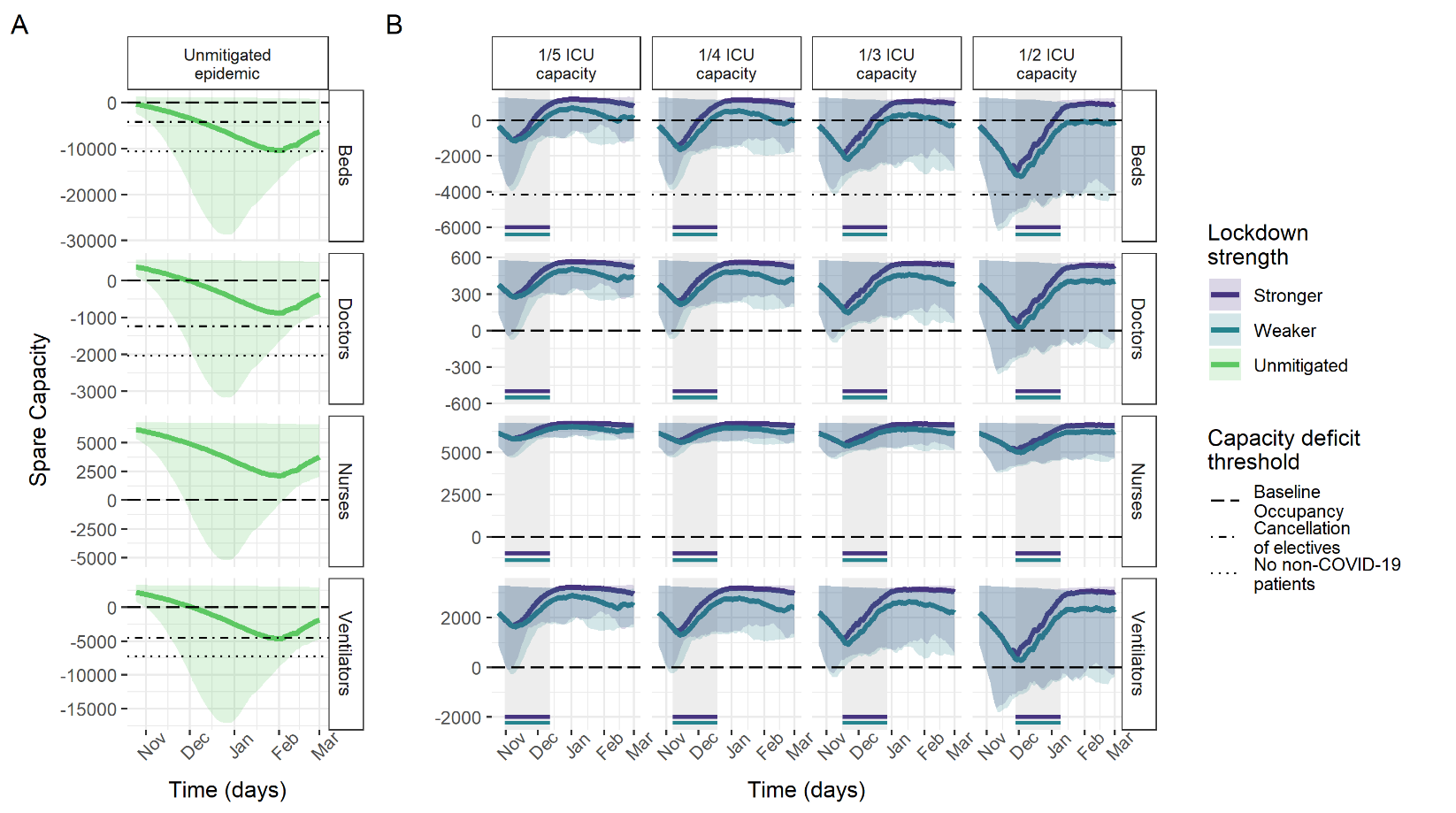


**Supplementary Figure S8: Spare capacity estimates (median; 95% credible intervals) for France.** (A) The unmitigated scenario. (B) The four reactive lockdown scenarios under two different suppression levels (stronger: lockdown effective reproduction number (R_t_) = 0.58; weaker: lockdown R_t_ = 0.8) and specified lockdown length of six weeks. Grey shaded areas indicate periods in which lockdowns are implemented, with horizontal coloured lines indicating the corresponding lockdown strength under which this was triggered. The dashed line (spare capacity = 0) indicates the threshold between positive spare capacity and a deficit in capacity. The dot-dashed and dotted lines indicate an effective reduction in this threshold owing to the cancellation of elective surgery and the removal of all non-coronavirus disease 2019 (non-COVID-19) patients respectively, allowing the reallocation of resources to COVID-19 patients.


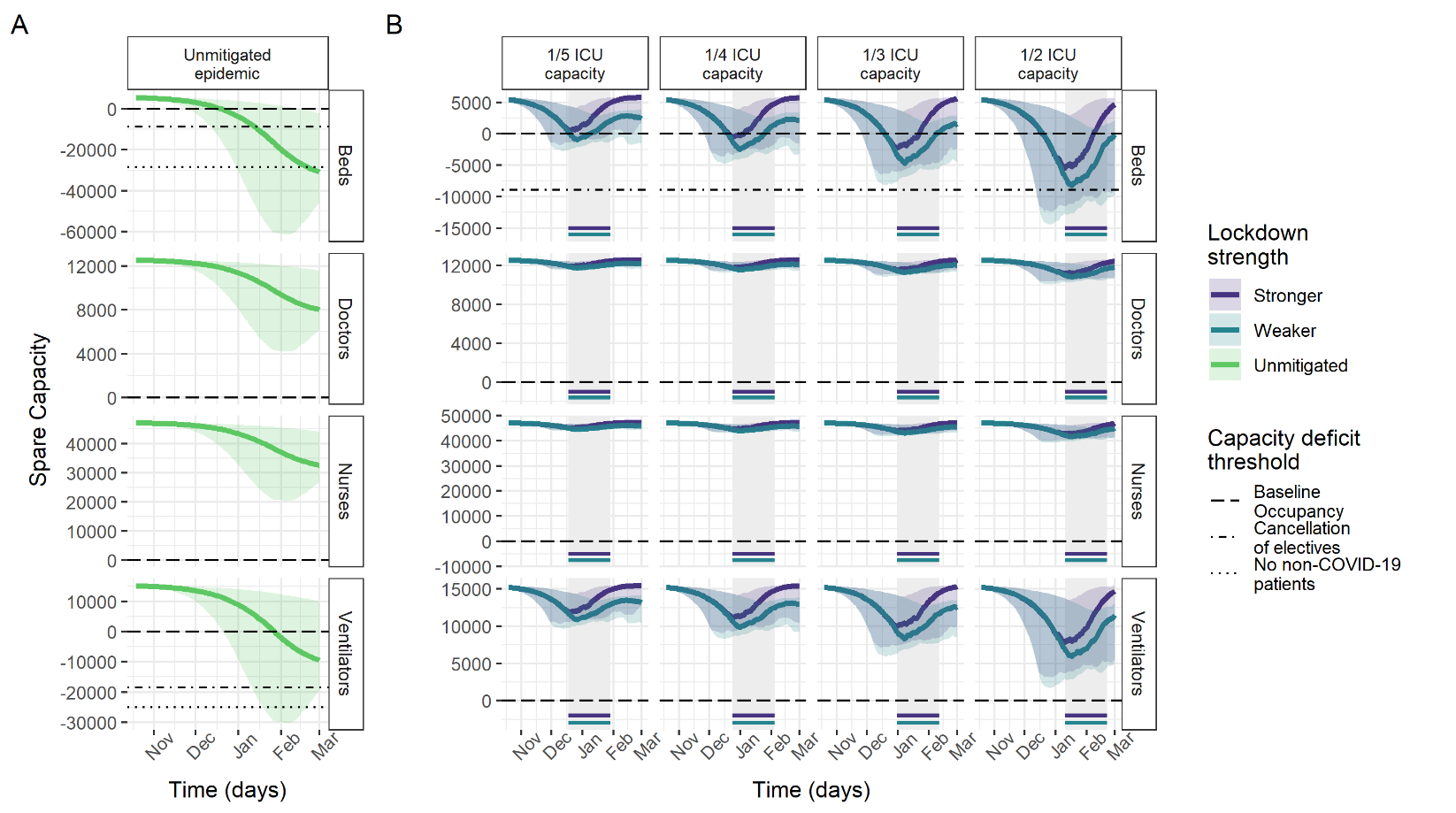


**Supplementary Figure S9: Spare capacity estimates (median; 95% credible intervals) for Germany.** (A) The unmitigated scenario. (B) The four reactive lockdown scenarios under two different suppression levels (stronger: lockdown effective reproduction number (R_t_) = 0.35; weaker: lockdown R_t_ = 0.8) and specified lockdown length of six weeks. Grey shaded areas indicate periods in which lockdowns are implemented, with horizontal coloured lines indicating the corresponding lockdown strength under which this was triggered. The dashed line (spare capacity = 0) indicates the threshold between positive spare capacity and a deficit in capacity. The dot-dashed and dotted lines indicate an effective reduction in this threshold owing to the cancellation of elective surgery and the removal of all non-coronavirus disease 2019 (non-COVID-19) patients respectively, allowing the reallocation of resources to COVID-19 patients.


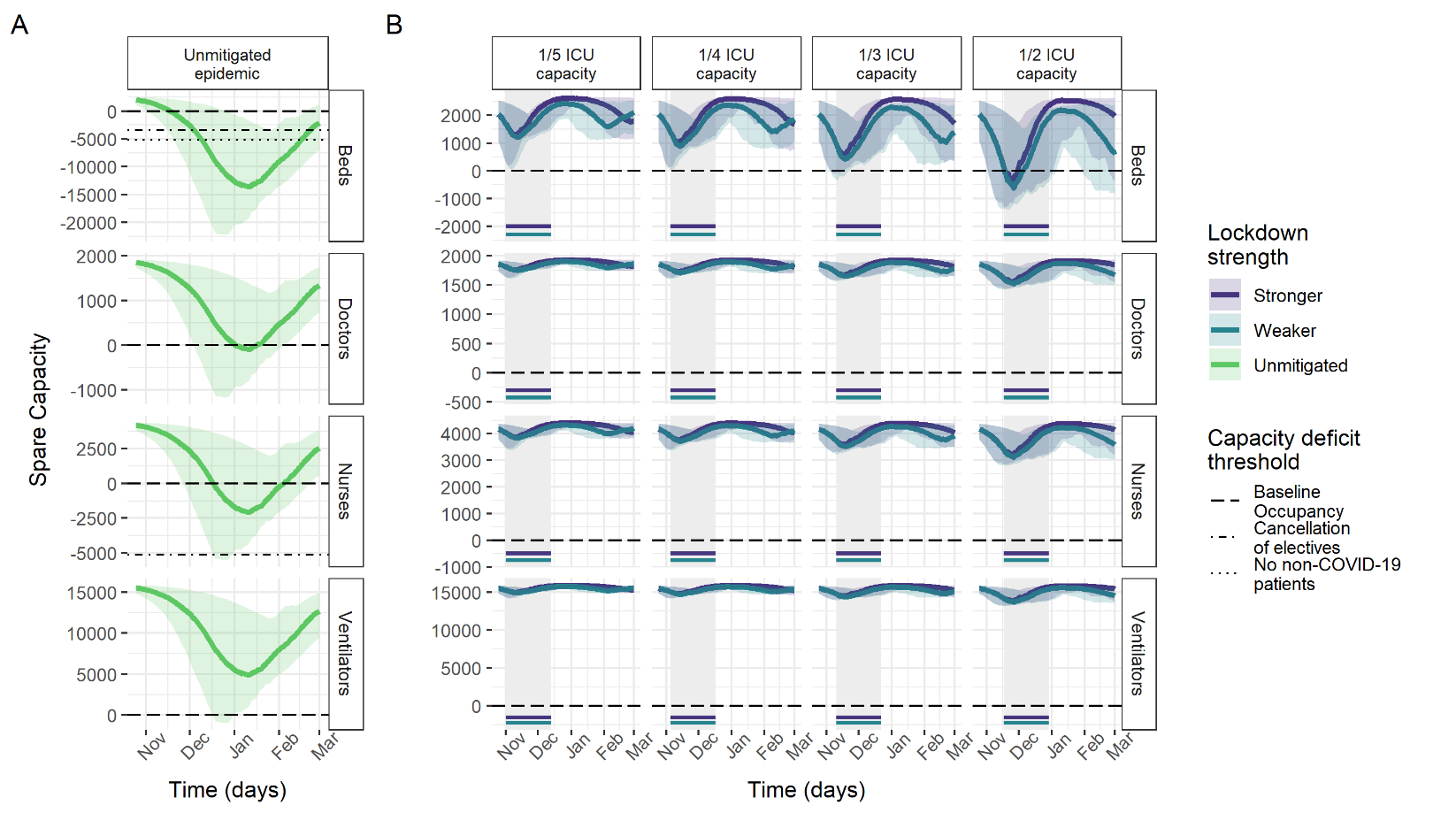


**Supplementary Figure S10: Spare capacity estimates (median; 95% credible intervals) for Italy.** (A) The unmitigated scenario. (B) The four reactive lockdown scenarios under two different suppression levels (stronger: lockdown effective reproduction number (R_t_) = 0.6; weaker: lockdown R_t_ = 0.8) and specified lockdown length of six weeks. Grey shaded areas indicate periods in which lockdowns are implemented, with horizontal coloured lines indicating the corresponding lockdown strength under which this was triggered. The dashed line (spare capacity = 0) indicates the threshold between positive spare capacity and a deficit in capacity. The dot-dashed and dotted lines indicate an effective reduction in this threshold owing to the cancellation of elective surgery and the removal of all non-coronavirus disease 2019 (non-COVID-19) patients respectively, allowing the reallocation of resources to COVID-19 patients.

**Supplementary Table S3: Median estimated maximum capacity deficit and number of days in deficit with 95% credible intervals for each country and capacity resource.** The unmitigated and reactive lockdown scenarios under two suppression levels (stronger^a^: lockdown effective reproduction number (R_t_) at levels estimated during first peak; weaker: lockdown R_t_ =0·8) are presented relative to baseline occupancy. Lockdown periods are specified to last for six weeks.

| Country | Resource | Result | Stronger lockdown^a^ | | | | Weaker lockdown | | | |
| --- | --- | --- | --- | --- | --- | --- | --- | --- | --- | --- |
|  |  |  | 1/5 ICU capacity | 1/4 ICU capacity | 1/3 ICU capacity | 1/2 ICU capacity | 1/5 ICU capacity | 1/4 ICU capacity | 1/3 ICU capacity | 1/2 ICU capacity |
| France | Beds | Maximum capacity deficit | 1205  (0-3711) | 1812  (0-3636) | 2799  (0-3899) | 4699  (0-6065) | 1300  (0-3966) | 1895  (0-3901) | 2905  (0-4160) | 4838  (0-6257) |
|  |  | Time in deficit (days) | *36*  *(0-64)* | *43*  *(0-69)* | *55*  *(0-81)* | *70*  *(0-111)* | *54*  *(0-93)* | *66*  *(0-108)* | *76*  *(0-112)* | *91*  *(0-127)* |
|  | Doctors | Maximum capacity deficit | 0  (0-46) | 0  (0-37) | 0  (0-70) | 170  (0-340) | 0  (0-78) | 0  (0-70) | 0  (0-102) | 187  (0-365) |
|  |  | Time in deficit (days) | *0*  *(0-10)* | *0*  *(0-9)* | *0*  *(0-13)* | *22*  *(0-28)* | *0*  *(0-15)* | *0*  *(0-15)* | *0*  *(0-18)* | *27*  *(0-55)* |
|  | Nurses | Maximum capacity deficit | 0^b^ | 0^b^ | 0^b^ | 0^b^ | 0^b^ | 0^b^ | 0^b^ | 0^b^ |
|  |  | Time in deficit (days) | *0^b^* | *0^b^* | *0^b^* | *0^b^* | *0^b^* | *0^b^* | *0^b^* | *0^b^* |
|  | Ventilators | Maximum capacity deficit | 0  (0-111) | 0  (0-60) | 0  (0-239) | 783  (0-1711) | 0  (0-284) | 0  (0-240) | 0  (0-416) | 877  (0-1842) |
|  |  | Time in deficit (days) | *0*  *(0-6)* | *0*  *(0-4)* | *0*  *(0-8)* | *20*  *(0-24)* | *0*  *(0-11)* | *0*  *(0-12)* | *0*  *(0-14)* | *24*  *(0-55)* |
| Germany | Beds | Maximum capacity deficit | 1089  (201-1938) | 2769  (1749-3812) | 5490  (1991-6975) | 10,714  (1991-13,088) | 1615  (554-2868) | 3459  (1983-4858) | 6311  (1991-8471) | 11,589  (1991-14,839) |
|  |  | Time in deficit (days) | *14*  *(6-17)* | *24*  *(16-26)* | *36*  *(16-40)* | *51*  *(16-57)* | *26*  *(12-53)* | *38*  *(15-61)* | *52*  *(16-70)* | *68*  *(16-79)* |
|  | Doctors | Maximum capacity deficit | 0^b^ | 0^b^ | 0^b^ | 0^b^ | 0^b^ | 0^b^ | 0^b^ | 0^b^ |
|  |  | Time in deficit (days) | *0^b^* | *0^b^* | *0^b^* | *0^b^* | *0^b^* | *0^b^* | *0^b^* | *0^b^* |
|  | Nurses | Maximum capacity deficit | 0^b^ | 0^b^ | 0^b^ | 0^b^ | 0^b^ | 0^b^ | 0^b^ | 0^b^ |
|  |  | Time in deficit (days) | *0^b^* | *0^b^* | *0^b^* | *0^b^* | *0^b^* | *0^b^* | *0^b^* | *0^b^* |
| Germany | Ventilators | Maximum capacity deficit | 0^b^ | 0^b^ | 0^b^ | 0^b^ | 0^b^ | 0^b^ | 0^b^ | 0^b^ |
|  |  | Time in deficit (days) | *0^b^* | *0^b^* | *0^b^* | *0^b^* | *0^b^* | *0^b^* | *0^b^* | *0^b^* |
| Italy | Beds | Maximum capacity deficit | 0^b^ | 0^b^ | *0*  *(0-182)* | 889  (394-1374) | 0^b^ | 0^b^ | 0  (0-381) | 1039  (390-1568) |
|  |  | Time in deficit (days) | *0^b^* | *0^b^* | *0*  *(0-8)* | *17*  *(12-26)* | *0^b^* | *0^b^* | *0*  *(0-12)* | *20*  *(13-43)* |
|  | Doctors | Maximum capacity deficit | 0^b^ | 0^b^ | 0^b^ | 0^b^ | 0^b^ | 0^b^ | 0^b^ | 0^b^ |
|  |  | Time in deficit (days) | *0^b^* | *0^b^* | *0^b^* | *0^b^* | *0^b^* | *0^b^* | *0^b^* | *0^b^* |
|  | Nurses | Maximum capacity deficit | 0^b^ | 0^b^ | 0^b^ | 0^b^ | 0^b^ | 0^b^ | 0^b^ | 0^b^ |
|  |  | Time in deficit (days) | *0^b^* | *0^b^* | *0^b^* | *0^b^* | *0^b^* | *0^b^* | *0^b^* | *0^b^* |
|  | Ventilators | Maximum capacity deficit | 0^b^ | 0^b^ | 0^b^ | 0^b^ | 0^b^ | 0^b^ | 0^b^ | 0^b^ |
|  |  | Time in deficit (days) | *0^b^* | *0^b^* | *0^b^* | *0^b^* | *0^b^* | *0^b^* | *0^b^* | *0^b^* |

^a^ France: R_t_ = 0.58; Germany: R_t_ = 0.35; Italy: R_t_ = 0.6.

^b^ No deficits projected under any of the 100 simulation replicates.

### Effect of varying trigger thresholds and duration on the impact and time in lockdown under weaker level of suppression

Supplementary Figure S11 shows the effect of varying trigger thresholds and lockdown duration on the impact and time in lockdown under the weaker level of suppression (effective reproduction number (R_t_) = 0.8). This is analogous to Figure 5.


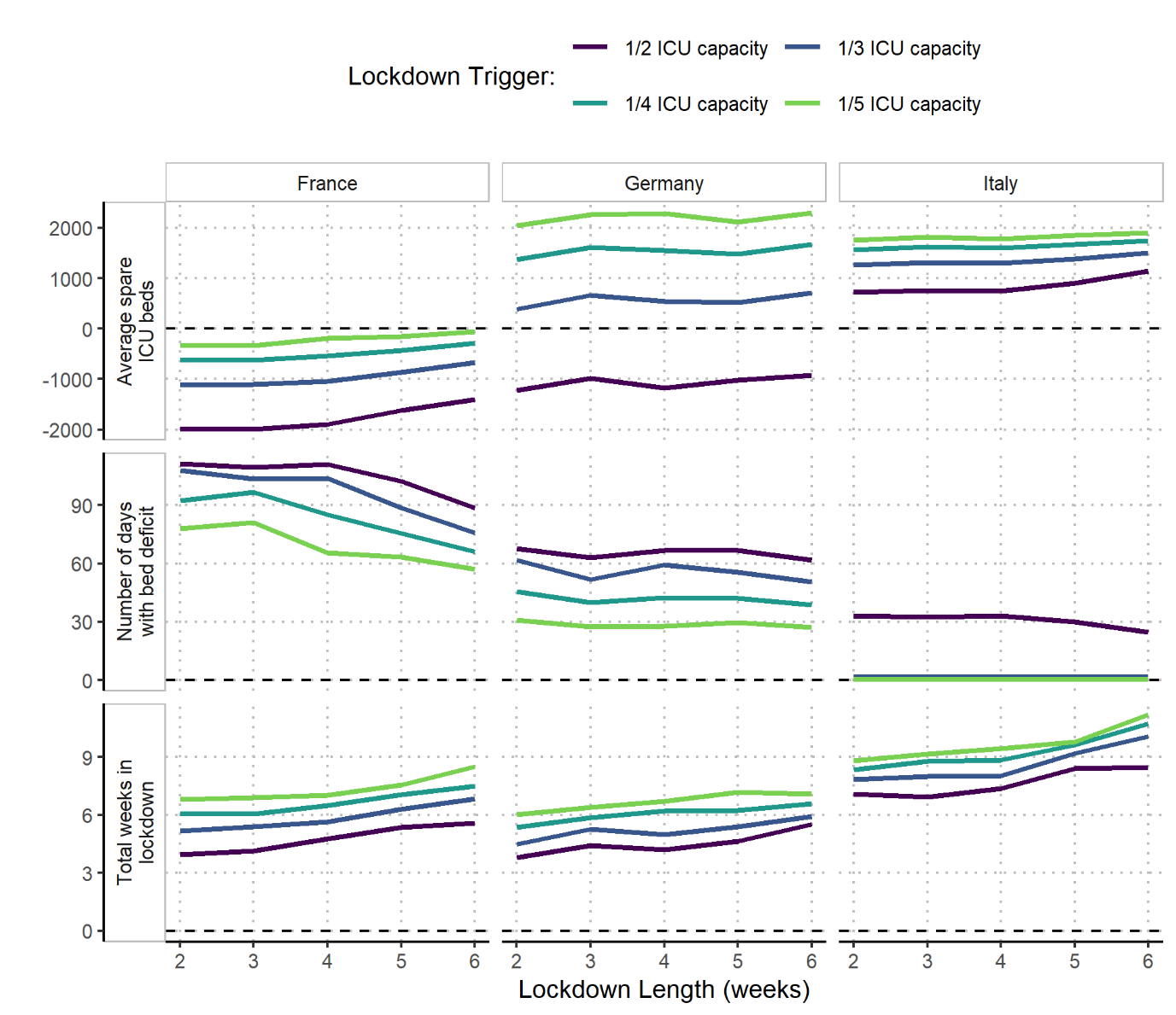


**Supplementary Figure S11: Impact of the duration and timing of lockdowns on spare capacity of intensive care unit (ICU) beds.** The effect of lockdown length on the average spare capacity of ICU beds; the number of days with a deficit in ICU beds and the total number of days spent in lockdown is shown for France, Germany and Italy under the weaker suppression scenarios (effective reproduction number (R_t_) = 0.8). For each plot the median of 100 simulation repetitions over the projection period (25^th^ October 2020 – March 2021) is shown.

### Scheduled lockdown scenarios

Supplementary Figures S12-S14 show scenarios in which lockdowns are fixed at pre-determined time points under the two levels of suppression for France, Germany, and Italy, respectively.


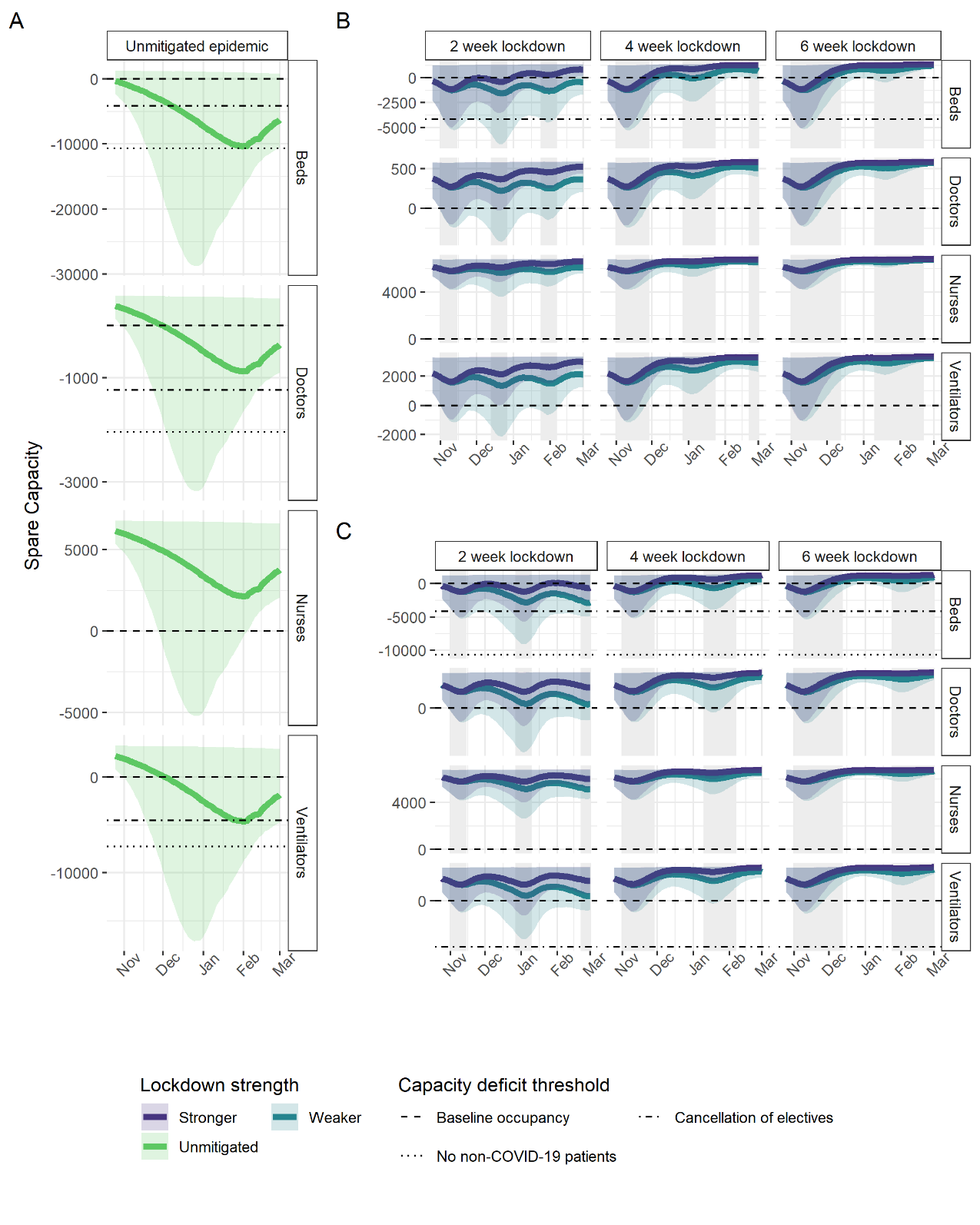


**Supplementary Figure S12: Spare capacity estimates (median; 95% credible intervals) for France.** (A) The unmitigated scenario. (B) Scheduled lockdown scenarios with four weeks between lockdowns under two different suppression levels (stronger: lockdown effective reproduction number (R_t_) = 0.58; weaker: lockdown R_t_ = 0.8). (C) Scheduled lockdown scenarios with four weeks between lockdowns under two different suppression levels (stronger: lockdown R_t_ = 0.58; weaker: lockdown R_t_ = 0.8). Grey shaded areas indicate periods of lockdown. The dashed line (spare capacity = 0) indicates the threshold between positive spare capacity and a deficit in capacity. The dot-dashed and dotted lines indicate an effective reduction in this threshold owing to the cancellation of elective surgery and the removal of all non-coronavirus disease 2019 (non-COVID-19) patients respectively, allowing the reallocation of resources to COVID-19 patients.


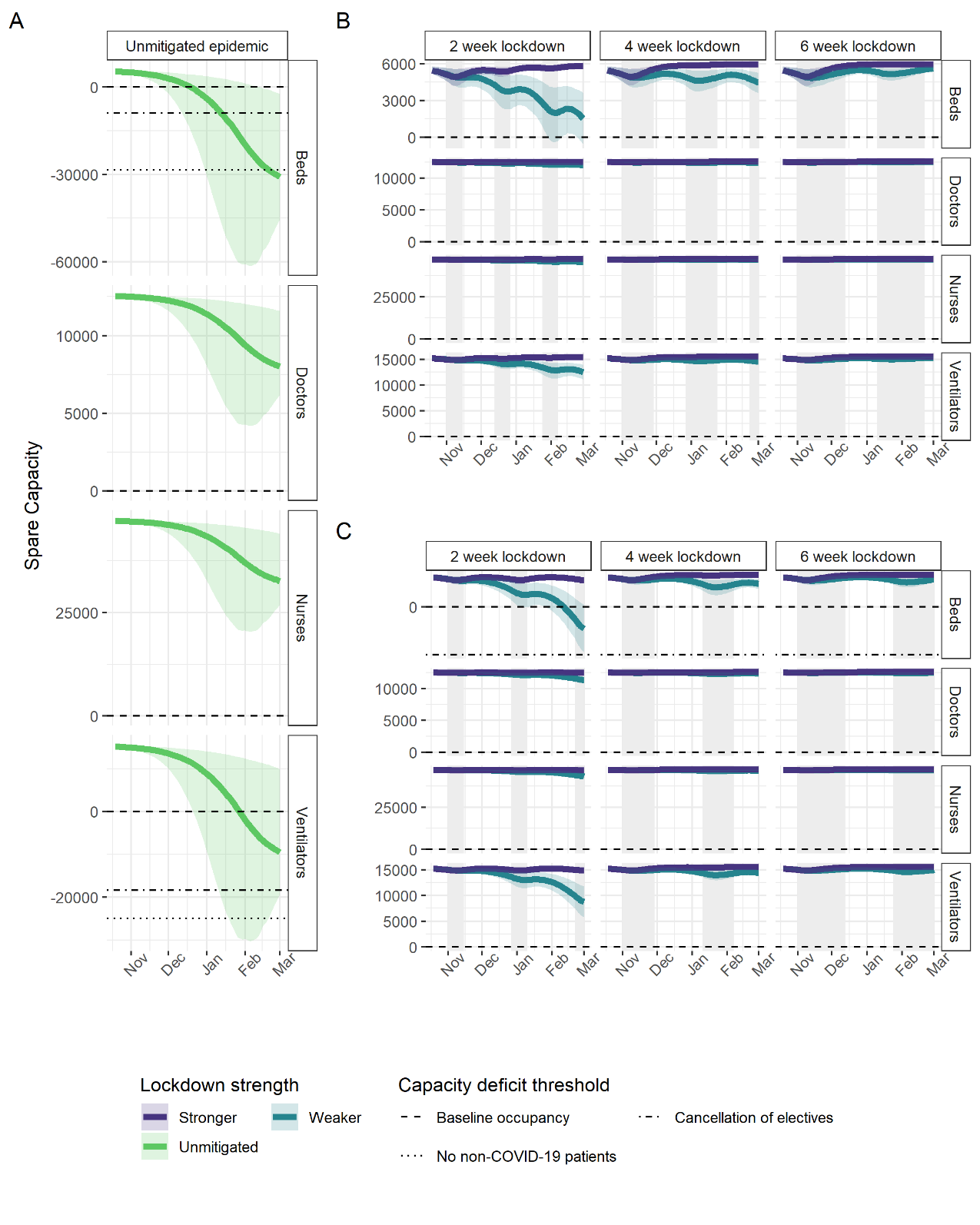
**Supplementary Figure S13: Spare capacity estimates (median; 95% credible intervals) for Germany.** (A) The unmitigated scenario. (B) Scheduled lockdown scenarios with four weeks between lockdowns under two different suppression levels (stronger: lockdown effective reproduction number (R_t_) = 0.35; weaker: lockdown R_t_ = 0.8). (C) Scheduled lockdown scenarios with four weeks between lockdowns under two different suppression levels (stronger: lockdown R_t_ = 0.35; weaker: lockdown R_t_ = 0.8). Grey shaded areas indicate periods of lockdown. The dashed line (spare capacity = 0) indicates the threshold between positive spare capacity and a deficit in capacity. The dot-dashed and dotted lines indicate an effective reduction in this threshold owing to the cancellation of elective surgery and the removal of all non-coronavirus disease 2019 (non-COVID-19) patients respectively, allowing the reallocation of resources to COVID-19 patients.


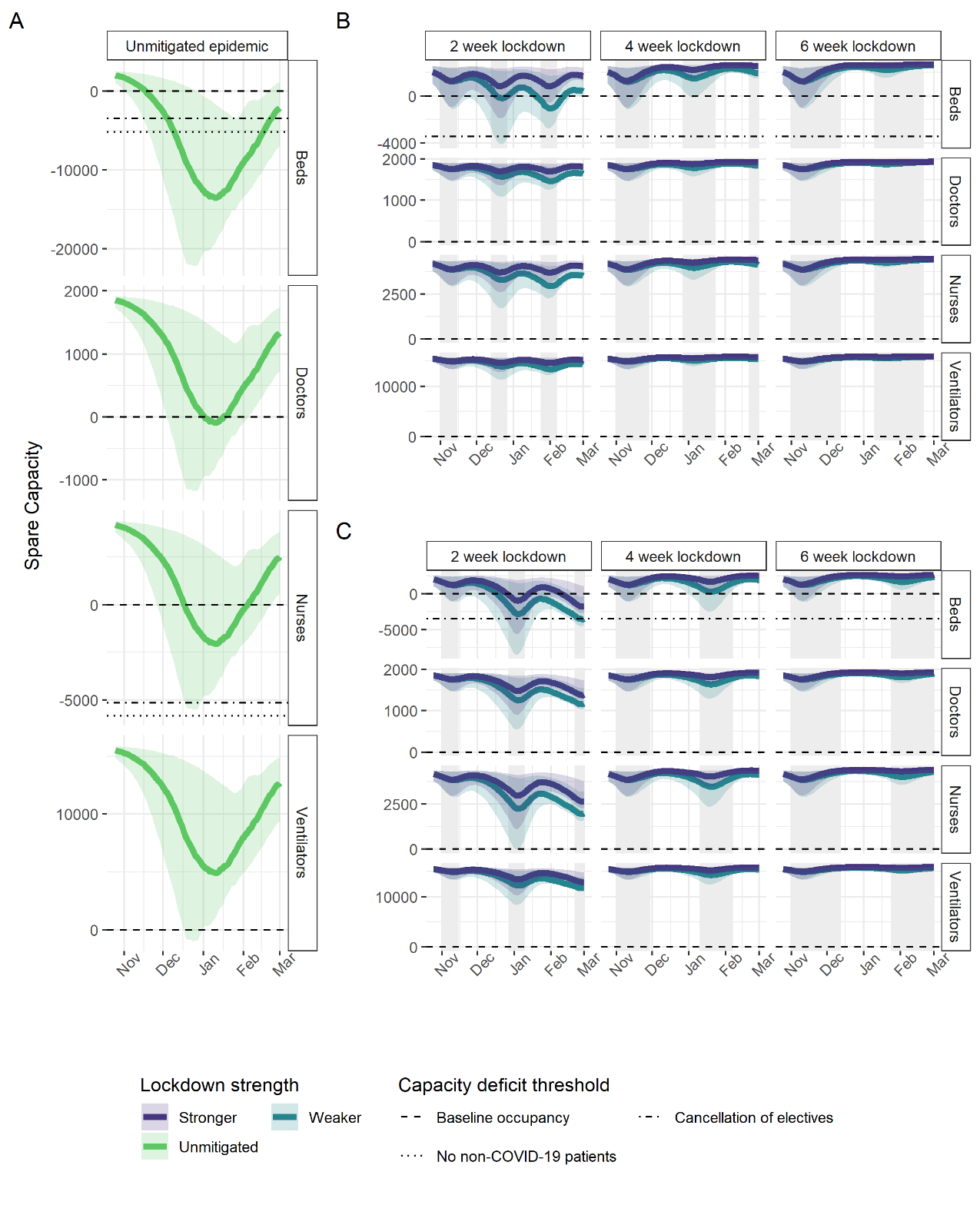
**Supplementary Figure S14: Spare capacity estimates (median; 95% credible intervals) for Italy.** (A) The unmitigated scenario. (B) Scheduled lockdown scenarios with four weeks between lockdowns under two different suppression levels (stronger: lockdown effective reproduction number (R_t_) = 0.6; weaker: lockdown R_t_ = 0.8). (C) Scheduled lockdown scenarios with four weeks between lockdowns under two different suppression levels (stronger: lockdown R_t_ = 0.6; weaker: lockdown R_t_ = 0.8). Grey shaded areas indicate periods of lockdown. The dashed line (spare capacity = 0) indicates the threshold between positive spare capacity and a deficit in capacity. The dot-dashed and dotted lines indicate an effective reduction in this threshold owing to the cancellation of elective surgery and the removal of all non-coronavirus disease 2019 (non-COVID-19) patients respectively, allowing the reallocation of resources to COVID-19 patients.

### Strategies to mitigate capacity deficits

The multifaceted nature of our model and subsequent analysis presents a unique opportunity to discuss the way in which different types of interventions can be combined in order to alleviate strain on ICUs over winter 2020/21.

First, the number of COVID-19 patients can be reduced by non-pharmaceutical interventions (NPIs), such as lockdowns and physical distancing, as demonstrated in this study. This strategy was widely deployed at the beginning of the epidemic and has been recently reintroduced in the countries under consideration here.^4–7^ Whilst lockdowns have proved to be effective in controlling COVID-19 epidemics across European settings,^4^ their wider, indirect impacts (on the economy, children’s education and broader population health^8^) risk inducing a public health and economic crisis larger than the one it aims to supress. Although reactive lockdowns can be triggered based on clear, logical criteria, they are likely to be disruptive and difficult to implement as they require accurate, timely data and there is uncertainty at the time of implementation in what their duration will be. Additionally, they still introduce two to eight weeks of lockdowns depending on the country. Our research suggests that scheduled lockdowns, with pre-specified start and end dates can also successfully overcome potential capacity deficits but with a greater amount of time in lockdown (5-12 weeks) (Supplementary Figures S12-S14). Such a strategy may be less socially disruptive by removing some uncertainty and may prove an effective strategy in countries in which fragmented health systems may lead to delays in implementing reactive lockdown strategies and where data on hospital occupancy is poor. However, we note that the success of such lockdowns is much more dependent on ensuring a stronger level of suppression. Furthermore, the efficacy of future lockdowns across different countries is uncertain. On the one hand, the experience with lockdowns during the first wave may help countries to implement them more effectively going forward. On the other hand, there is evidence to suggest that the population may be less likely to adhere strictly to control policies with each successive lockdown.^9^ This makes the long-term tenability of this strategy unclear. Nevertheless, in this study even weaker reactive lockdown scenarios led to substantial reductions in ICU bed shortages compared to the unmitigated scenario.

Second, capacity deficits may be partially managed by reducing the number of non-COVID-19 patients admitted to ICU. This is primarily achieved through the cancellation of elective surgery,^10^ although our results suggest that on its own this would not be sufficient to address the deficits in ICU bed capacity in France, Germany, and Italy over the winter season. Moreover, this policy pushes the healthcare burden onto a different group of patients, possibly resulting in excess morbidity and mortality.^11^ National-level triaging criteria for the allocation of ICU resources in the event of demand outstripping supply were also introduced in some places, for example in Italy.^12^ However, this raises complex ethical issues. Ultimately, under such strategies the burden is redistributed rather than alleviated and it is unclear whether the short-term benefits will outweigh the long-term costs.

Finally, as an alternative to managing patient demand, ICU capacity can be increased through supply-side hospital provision interventions. This was a common approach at the beginning of the outbreak^10^ and are often most effective when implemented simultaneously.^11^ For example, countries achieved increases in bed numbers by setting up surge capacity (such as field hospitals and requisitioning the use of private healthcare facilities) to treat patients with complex care needs.^13–15^ Our results suggest that such measures to increase bed supply may still be needed to address small remaining deficits even with implementation of a lockdown but, critically, could prevent the need to cancel elective surgery. Additionally, the results also suggest that generally there is a sufficient capacity of staff and ventilators to fully operationalise any additional required surge beds.^11,16^ Although often expensive and with logistical challenges ahead of implementation, it is likely that hospital provision interventions will continue to be a vitally important tool in terms of expanding ICU capacity to ensure the provision of care to all patients during the pandemic this winter.

### Supplementary Material References

1 Google. COVID-19 Community Mobility Reports. 2020. https://www.google.com/covid19/mobility/ (accessed Oct 25, 2020).

2 European Centre for Disease Prevention and Control. Publications & Data. 2020. https://www.ecdc.europa.eu/en/publications-data/download-data-hospital-and-icu-admission-rates-and-current-occupancy-covid-19) (accessed Oct 25, 2020).

3 Robert Koch Institut. Aktueller Lage-/Situationsbericht des RKI zu COVID-19. 2020. https://www.rki.de/DE/Content/InfAZ/N/Neuartiges_Coronavirus/Situationsberichte/Gesamt.html (accessed Aug 28, 2020).

4 Flaxman S, Mishra S, Gandy A, *et al.* Estimating the effects of non-pharmaceutical interventions on COVID-19 in Europe. *Nature* 2020; **584**: 257–61.

5 Gouvernement de la République française. Informations Coronavirus. 2020. https://www.gouvernement.fr/en/coronavirus-covid-19 (accessed Nov 9, 2020).

6 Bundesregierung. Bundesrat stimmt Gesetzespaketen zur Unterstützung des Gesundheitswesens bei der Bewältigung der Corona-Epidemie zu. 2020. https://www.bundesgesundheitsministerium.de/presse/pressemitteilungen/2020/1-quartal/corona-gesetzespaket-im-bundesrat.html (accessed April 29, 2020).

7 Governo Italiano. Il Decreto Ristori. 2020. http://www.governo.it/it/approfondimento/il-decreto-ristori/15550 (accessed Nov 9, 2020).

8 Han E, Tan MMJ, Turk E, *et al.* Lessons learnt from easing COVID-19 restrictions: an analysis of countries and regions in Asia Pacific and Europe. *The Lancet* 2020; : S0140673620320079.

9 World Health Organization Regional Office for Europe. Pandemic fatigue Reinvigorating the public to prevent COVID-19. 2020 https://apps.who.int/iris/bitstream/handle/10665/335820/WHO-EURO-2020-1160-40906-55390-eng.pdf (accessed Oct 8, 2020).

10 Christen P, D’Aeth JC, Løchen A, *et al.* The J-IDEA Pandemic Planner: A Framework for Implementing Hospital Provision Interventions During the COVID-19 Pandemic. *Medical Care* 2021; **Publish Ahead of Print**. DOI:10.1097/MLR.0000000000001502.

11 McCabe R, Schmit N, Christen P, *et al.* Adapting hospital capacity to meet changing demands during the COVID-19 pandemic. *BMC Med* 2020; **18**: 329.

12 Vergano M, Bertolini G, Giannini A, *et al.* Raccomandazioni di etica clinica per l’ammissione a trattamenti intensivi e per la loro sospensione, in condizioni eccezionali di squilibrio tra necessità e risorse disponibili. 2020. http://www.siaarti.it/SiteAssets/News/COVID19%20-%20documenti%20SIAARTI/SIAARTI%20-%20Covid19%20-%20Raccomandazioni%20di%20etica%20clinica.pdf.

13 Or Z, Gandre C. COVID-19 Health Policy Response Monitor: Policy responses for France. COVID-19 Health System Response Monitor. 2020. https://www.covid19healthsystem.org/countries/france/countrypage.aspx (accessed Sept 1, 2020).

14 Winklemann J, Reichebner C. COVID-19 Health Policy Response Monitor: Policy responses for Germany. COVID-19 Health System Response Monitor. 2020; published online March 31. https://www.covid19healthsystem.org/countries/germany/livinghit.aspx?Section=2.2%20Workforce&Type=Section (accessed Sept 1, 2020).

15 Fattore G, de Belvis AG, Ricciardi W, *et al.* COVID-19 Health Policy Response Monitor: Policy responses for Italy. COVID-19 Health System Response Monitor. 2020. https://www.covid19healthsystem.org/countries/italy/countrypage.aspx (accessed Sept 1, 2020).

16 Nelson B. Too little or too much? Missing the Goldilocks zone of hospital capacity during covid-19. *BMJ* 2020; **369**. DOI:10.1136/bmj.m2332.
